# Unraveling the Atomic‐Level Manipulation Mechanism of Li_2_S Redox Kinetics via Electron‐Donor Doping for Designing High‐Volumetric‐Energy‐Density, Lean‐Electrolyte Lithium–Sulfur Batteries

**DOI:** 10.1002/advs.202204192

**Published:** 2022-10-06

**Authors:** Jiongwei Shan, Wei Wang, Bing Zhang, Xinying Wang, Weiliang Zhou, Liguo Yue, Yunyong Li

**Affiliations:** ^1^ School of Materials and Energy Guangdong University of Technology No. 100 Waihuan Xi Road, Guangzhou Higher Education Mega Center Guangzhou 510006 China

**Keywords:** Cu‐doped CoP/MXene, dense sulfur cathodes, Li_2_S redox kinetics, lithium–sulfur batteries, volumetric capacity

## Abstract

Designing dense thick sulfur cathodes to gain high‐volumetric/areal‐capacity lithium–sulfur batteries (LSBs) in lean electrolytes is extremely desired. Nevertheless, the severe Li_2_S clogging and unclear mechanism seriously hinder its development. Herein, an integrated strategy is developed to manipulate Li_2_S redox kinetics of CoP/MXene catalyst via electron‐donor Cu doping. Meanwhile a dense S/Cu_0.1_Co_0.9_P/MXene cathode (density = 1.95 g cm^−3^) is constructed, which presents a large volumetric capacity of 1664 Ah L^−1^ (routine electrolyte) and a high areal capacity of ≈8.3 mAh cm^−2^ (lean electrolyte of 5.0 µL mg_s_^−1^) at 0.1 C. Systematical thermodynamics, kinetics, and theoretical simulation confirm that electron‐donor Cu doping induces the charge accumulation of Co atoms to form more chemical bonding with polysulfides, whereas weakens Co—S bonding energy and generates abundant lattice vacancies and active sites to facilitate the diffusion and catalysis of polysulfides/Li_2_S on electrocatalyst surface, thereby decreasing the diffusion energy barrier and activation energy of Li_2_S nucleation and dissolution, boosting Li_2_S redox kinetics, and inhibiting shuttling in the dense thick sulfur cathode. This work deeply understands the atomic‐level manipulation mechanism of Li_2_S redox kinetics and provides dependable principles for designing high‐volumetric‐energy‐density, lean‐electrolyte LSBs through integrating bidirectional electro‐catalysts with manipulated Li_2_S redox and dense‐sulfur engineering.

## Graphical abstract

The authors develop an integrated strategy to manipulate Li_2_S redox kinetics of CoP/MXene catalyst via electron‐donor Cu doping and meanwhile construct a dense S/Cu_0.1_Co_0.9_P/MXene cathode, which presents large volumetric capacity of 1664 Ah L^−1^ and high areal capacity of ≈8.3 mAh cm^−2^ (lean electrolyte) at 0.1 C, and its atomic‐level manipulation mechanism of Li_2_S redox kinetics is uncovered.

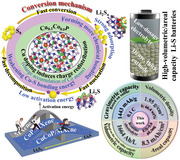

## 1 Introduction

The fast development of mobile electronic devices and electric vehicles urgently need to develop a high energy storage system in a limited space.^[^ ^1^, ^2^ ^]^ Lithium–sulfur batteries (LSBs) have become the research focus of the potential energy storage device because of low cost, high specific capacity, and especially high theoretical energy density (≈2800 Wh L^−1^).^[^ ^3^, ^4^, ^5^ ^]^ Unfortunately, low sulfur loading, low sulfur content, low cathode density, and excess electrolyte lead to the actual volumetric energy density far less than 2800 Wh L^−1^.^[^ ^6^, ^7^, ^8^ ^]^ Hence, high compacted density, high sulfur loading, and content of sulfur cathode as well as lean electrolyte are preconditions to realize the high volumetric and areal capacity of LSBs.^[^ ^9^, ^10^, ^11^, ^12^ ^]^ Nevertheless, the high sulfur loading, sulfur content, and high cathode density can cause extensive polysulfide accumulation in electrolyte, thus extremely aggravating the polysulfide shuttling and the polarization of electrodes.^[^ ^13^, ^14^, ^15^ ^]^ In the reduction reaction process of sulfur species, fast conversion from insoluble S_8_ to soluble Li_2_S*_n_* (4 ≤ *n* ≤ 8, i.e., polysulfides) (S_8_ → Li_2_S*_n_*) and slow conversion from soluble Li_2_S*_n_* to insoluble Li_2_S (Li_2_S*_n_* → Li_2_S) are the main reason of severe polysulfide shuttling. Therefore, these differences of conversion rate can cause the increase of Li_2_S*_n_* content in electrolyte.^[^ ^16^, ^17^, ^18^ ^]^ In the oxidation reaction process of sulfur species, slow conversion from insoluble Li_2_S to soluble Li_2_S*_n_* (Li_2_S → Li_2_S*_n_*), Li_2_S are unevenly deposited on the electrode surface to form large‐size particles, thus reducing the utilization rate of sulfur and exacerbating electrochemical polarization.^[^ ^19^, ^20^, ^21^ ^]^ Hence, the Li_2_S redox kinetics are the rate‐determining step during cycling of LSBs.^[^ ^22^ ^]^

Therefore, improving the Li_2_S redox kinetics of high‐sulfur loading/content cathode with high compacted density are essential for realizing the high volumetric and areal capacity of LSBs in a low‐dosage electrolyte. A sulfur host is supposed to possess a superior catalytic activity, simultaneously owning a high conductivity and a specific surface area.^[^ ^23^, ^24^, ^25^ ^]^ Nowadays, 2D transition metal phosphides (TMPs) (e.g., CoP) have attracted continuous attention because of their good catalytic activity in promoting the conversions of polysulfides.^[^ ^26^, ^27^, ^28^ ^]^ Furthermore, TMPs exhibited a strong adsorption ability for polysulfides, good conductivity, and easily adjustable electronic structure.^[^ ^29^, ^30^, ^31^ ^]^ Although 2D TMPs have displayed great advantages in promoting the electrochemical conversion of polysulfides, their abundant edge active sites and the limited catalytic active sites of TMPs in the inertly basal plane hinder the catalytic conversion of Li_2_S.^[^ ^32^ ^]^ Therefore, it is considered an effective way to further improve the activity of the TMPs catalyst by adjusting the electronic structure using doping/defect engineering.^[^ ^33^, ^34^, ^35^ ^]^

Based on previous reports on doping/defect engineering in LSBs, it is expected to enhance the adsorption ability of TMPs for Li_2_S*_n_* and Li_2_S redox kinetics (Li_2_S nucleation/decomposition) via adjusting the catalytic efficiency and active sites of TMPs.^[^ ^29^ ^]^ Electron‐donor doping (e.g., Cu) could play the excellent catalytic behavior of active sites through optimizing the cationic activity of TMPs (high catalytic efficiency of the active site) and introducing vacancies to expose more active sites, which is expected to improve the catalytic performance of TMPs.^[^ ^36^, ^37^, ^38^ ^]^ However, promoting the redox kinetics of Li_2_S via electron‐donor doping simultaneously adjusting the atomic activity, electronic structure, and the number of active sites of the catalyst is rarely reported in LSBs. More importantly, how atomic activity affects the adsorption ability and catalytic activity of TMPs for polysulfides and Li_2_S redox is still lacking in‐depth understanding, particularly in a dense high‐sulfur‐loaded cathode.

Besides, pure nano‐TMPs (e.g., 2D CoP nanosheets) have high surface energy and are easy to agglomerate; generally, TMPs need to be coupled with highly conductive and large surface area substrates (e.g., MXene) to avoid agglomeration between them.^[^ ^39^, ^40^, ^41^ ^]^ The synergistic effect of MXene (high surface area and rich functional groups) and 2D CoP nanosheets is beneficial to enhance the capture ability for polysulfides and efficiently promote the redox kinetics of Li_2_S in LSBs.^[^ ^42^, ^43^, ^44^, ^45^ ^]^

**Figure 1:**
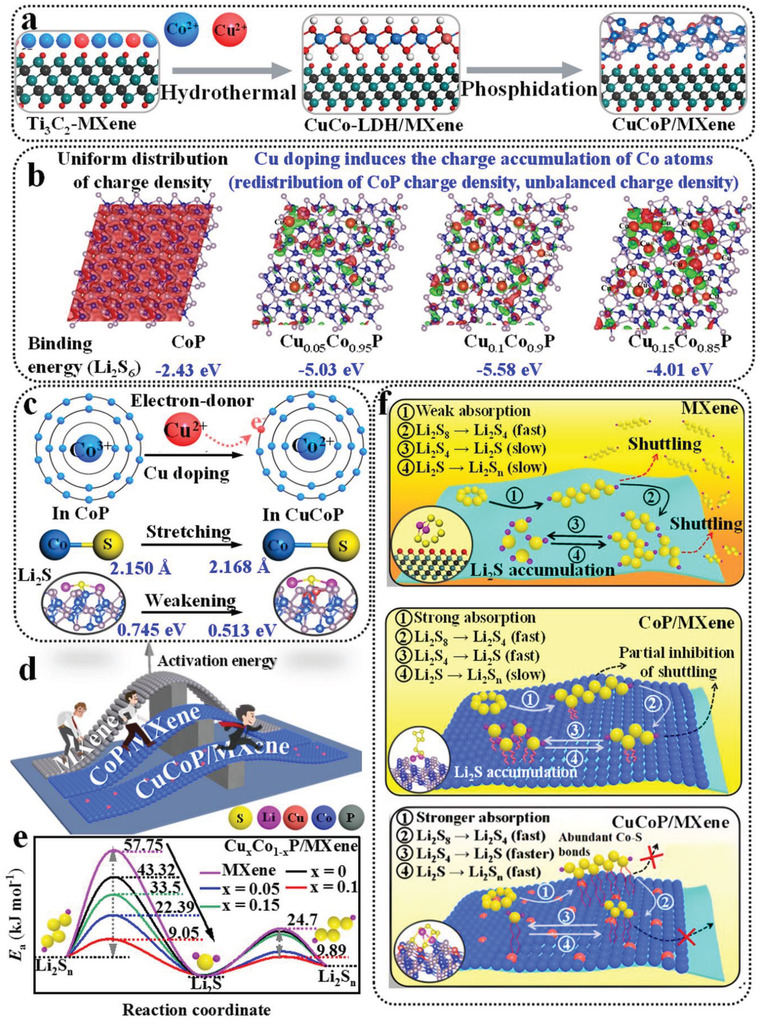
Scheme of the enhanced Li_2_S redox kinetic mechanism of electron‐donor Cu‐doped CoP/MXene (CuCoP/MXene) catalyst. a) Synthesis scheme of the CuCoP/MXene catalysts. b) The 3D charge densities of Cu*_x_* Co_1−_ *_x_* P (atomic ratio, *x* = 0, 0.05, 0.1, and 0.15) (isosurface level is set to 0.004 e Å^−3^) (red and green represent the electron accumulate and depletion regions, respectively). c) Schematic of electron transfer and bond length in CoP/MXene and CuCoP/MXene. d,e) *E*_a_ for polysulfides/Li_2_S conversion on CuCoP/MXene catalyst and pure MXene. f) Polysulfides/Li_2_S conversion on MXene, CoP/MXene, and CuCoP/MXene catalyst surfaces.

**Figure 2:**
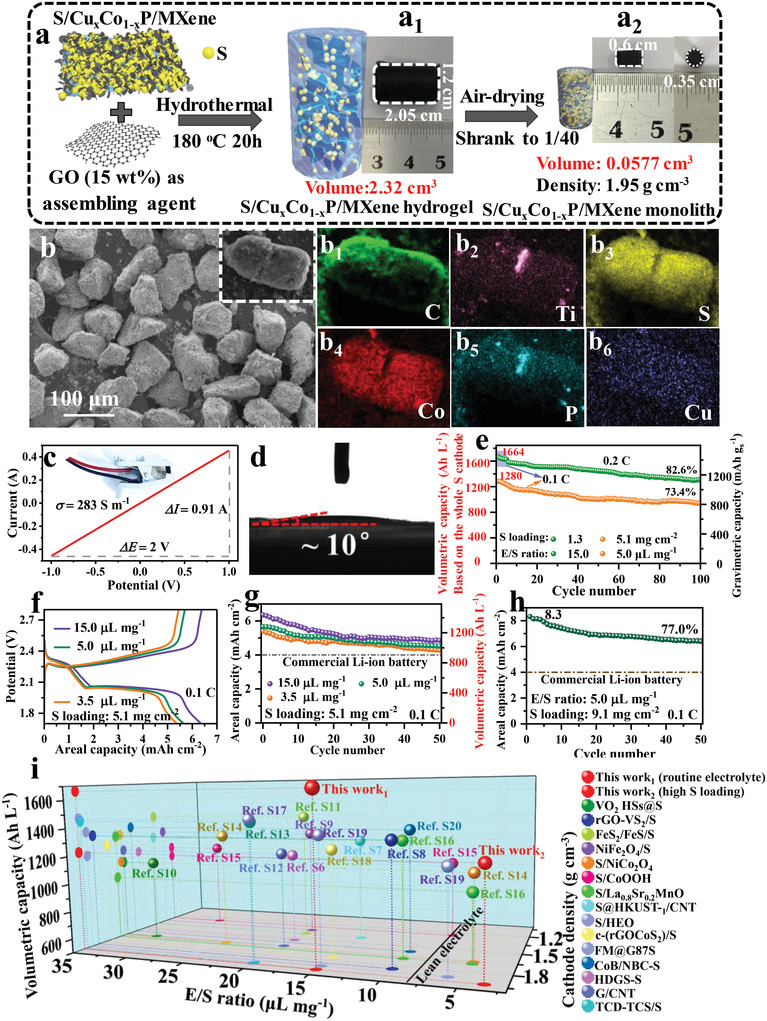
Electrochemical performance of dense S/Cu_0.1_Co_0.9_P/MXene cathode. a) Synthesis scheme of the dense S/Cu_0.1_Co_0.9_P/MXene monolith and its optical photos a1) before and a2) after air drying. b) SEM images and b_1_–b_6_) its elemental mapping of C, Ti, S, Co, P, and Cu. c) CV curve for the dense S/Cu_0.1_Co_0.9_P/MXene monolith (Inset is the testing device). d) Surface wetting of electrolyte droplets on the dense S/Cu_0.1_Co_0.9_P/MXene cathode. e) Cycling stability in a routine/low‐dosage electrolyte at S loading of 1.3/5.1 mg cm^−2^. f) GDC curves and g) areal and volumetric capacities with sulfur loading of 5.1 mg cm^−2^ under the E/S ratios of 3.5, 5.0, and 15.0 µL mg_s_^−1^. h) Areal capacity with sulfur loading of 9.1 mg cm^−2^ under the E/S ratios of 5.0 µL mg_s_^−1^. i) This work is compared with the recently reported advanced sulfur cathodes in volumetric capacity, cathode density, and E/S ratios.

In this work, we develop an integrated strategy to manipulate the Li_2_S redox kinetics of CoP/MXene bidirectional catalyst via electron‐donor Cu doping and meanwhile construct a dense sulfur cathode, thereby designing a dense S/CuCoP/MXene cathode, which acts as a model to understand the atomic‐level manipulation mechanism of Li_2_S redox kinetics and achieve high‐volumetric/areal‐capacity LSBs in lean‐electrolyte. Specifically, 2D few‐layer Cu‐doped CoP are in situ grown on MXene nanosheets (denoted as Cu*_x_* CoP_1−_ *_x_*/MXene) via a hydrothermal reaction and followed by phosphorization (see **Figure** **1**a and Figure S1a, Supporting Information), which acts as a highly efficient sulfur host (sulfur loading: ≈78.6 wt%) (Here, Cu*_x_* CoP_1−_ *_x_*/MXene catalysts with different Cu doping contents [*x* = 0, 0.05, 0.1, and 0.15] were investigated to acquire the optimal catalyst). And then, to gain superior volumetric performance of the practical LSBs, a dense S/Cu_0.1_Co_0.9_P/MXene cathode was fabricated through forming and shrinking the S/Cu_0.1_Co_0.9_P/MXene hydrogel via using a low content of graphene oxide (GO) (≈15 wt%) as the “assembling agent” (**Figure** **2**a). Here, the optimal S/Cu_0.1_Co_0.9_P/MXene cathode displays the largest gravimetric specific capacity and the most stable cycle performance. By integrating dense sulfur engineering, the dense S/Cu_0.1_Co_0.9_P/MXene cathode, with high density of 1.95 g cm^−3^ and high electrical conductivity of 283 S m^−1^, gives an ultrahigh volumetric capacity of 1664 Ah L^−1^ (based on the whole volume of S cathode) and gravimetric capacity of 1443 mAh g^−1^ (based on S) at 0.1 C, and superior cycling performance within 100 cycles (capacity retention rate = 82.6%) at 0.2 C in a routine electrolyte. Impressively, at a low electrolyte/sulfur (E/S) ratio of 5.0 µL mg_s_^−1^, the dense, thick sulfur cathode still retains a superior volumetric capacity (1280 Ah L^−1^) and a large areal capacity (≈8.3 mAh cm^−2^) at 0.1 C. Even at the lower E/S ratio of 3.5 µL mg_s_^−1^, its volumetric capacity still maintains at 1219 Ah L^−1^. Such ultrahigh volumetric capacity outperforms these recently reported advanced sulfur cathodes, particularly in the lean electrolyte (see Figure 2i and Table S2, Supporting Information).

Systematical investigations of density functional theory (DFT) calculation, kinetics, and thermodynamics clearly uncover the atomic‐level manipulation mechanism of Li_2_S redox kinetics via electron‐donor Cu doping, which originates from the following advantages: 1) electron‐donor Cu doping can induce the charge accumulation of Co and P atoms near Cu atoms, thus generating more unbalanced charge distribution areas to form more chemical bonds with polysulfides (more Co/Cu—S bonding), thereby enhancing the adsorption ability with polysulfides (enhanced from −2.43 to −5.58 eV); 2) electron‐donor Cu doping can easily make the strongly electronegative Co^3+^ (electronegativity = 15.26) in CoP converted into weakly electronegative Co^2+^ (electronegativity = 9.10) via seizing the extranuclear electrons of Cu atoms, so it weakens the Co—S bonding energy (lengthening the bond length of Co—S bonds from 2.150 to 2.169 Å), meanwhile generates abundant lattice vacancies and active sites, thereby facilitating the diffusion and catalysis of polysulfides/Li_2_S on the electrocatalyst surface, so decreasing the diffusion energy barrier and activation energy of Li_2_S nucleation and decomposition, and finally intrinsically boosting the Li_2_S redox kinetics; 3) the synergistic effect between the modulated CoP and Ti_3_C_2_‐MXene can effectively enhance the overall electron conduction, active area, and structural stability of the hybrid catalyst, thus endowing the catalyst satisfactory electrocatalytic activity and capture capability for polysulfides; 4) a dense sulfur structure can endow large volumetric performance, and the 3D hydrophilic MXene and graphene conductive networks can ensure good electrolyte permeation and efficient electron/ion transmission in a low‐dosage electrolyte. Therefore, this work, from the experimental and theoretical results, deeply understands the manipulating mechanism for Li_2_S redox kinetics via electron‐donor doping at the electronic and atomic level, and also provides a dependable design principle for lean‐electrolyte LSBs with high volumetric and areal capacity through integrating a dual‐directional electrocatalyst with manipulated Li_2_S redox kinetics and dense‐sulfur engineering.

## 2 Results and Discussion

### 2.1 Manipulating Mechanism Analysis for the Li_2_S/Polysulfides Redox Kinetics of CoP/MXene Catalyst via Electron‐Donor Cu Doping

**Figure 3:**
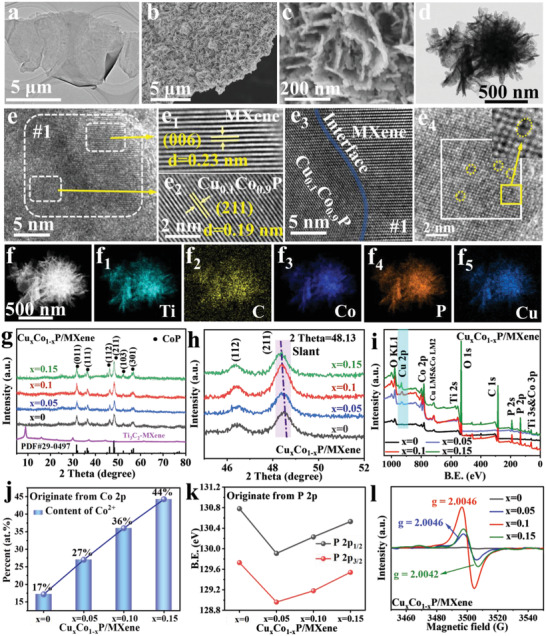
Physical characterization of Cu*_x_* Co_1−_ *_x_* P/MXene (*x* = 0, 0.05, 0.1, and 0.15) and MXene. a) TEM image of Ti_3_C_2_‐MXene. b,c) SEM, d) TEM, and e‐e_4_) HR‐TEM images of Cu_0.1_Co_0.9_P/MXene. f) STEM and the corresponding EDX elemental mappings of f_1_–f_5_) Ti, C, Co, P, and Cu in Cu_0.1_Co_0.9_P/MXene. g,h) XRD patterns of MXene and Cu*_x_* Co_1−_ *_x_* P/MXene. i) XPS survey spectra of Cu*_x_* Co_1−_ *_x_* P/MXene. j) The contents of Co^2+^ of Co 2p XPS spectra and k) the binding energies of P 2p_1/2_ and P 2p_3/2_ XPS spectra in Cu*_x_* Co_1−_ *_x_* P/MXene. l) EPR spectra of Cu*_x_* Co_1−_ *_x_* P/MXene.

The synthesis route of electron‐donor Cu doping CoP grown on MXene (CuCoP/MXene) is schematically illustrated in Figure 1a and Figure S1a, Supporting Information. A few‐layer Ti_3_C_2_‐MXene matrix with high conductivity and specific surface area was first synthesized (**Figure** **3**a and Figure S1b, Supporting Information) through a reported method and then the CuCo‐layered double hydroxides (CuCo‐LDH) grown on MXene was synthesized (Figures S1c and S2, Supporting Information) via hydrothermal reaction and finally converted to CuCoP/MXene (Figures S1d,e and S3, Supporting Information) through phosphorization. CoP/MXene with different Cu doping contents were also obtained by adjusting the ratio of Cu to Co atoms, which are labeled as Cu*_x_* Co_1−_ *_x_* P/MXene (*x* = 0, 0.05, 0.1, and 0.15). And the accurate content of Cu and Co elements in Cu*_x_* Co_1−_ *_x_* P/MXene with different Cu doping contents was further determined by inductively coupled plasma mass spectrometry, which was showed in Figure S5, Supporting Information, respectively. The actual content is near close to the theory.

To understand the roles of electron‐donor Cu doping engineering on Li_2_S/polysulfides conversion in LSBs, DFT calculation was carried out with CoP as the research object. First, the differences between the electron distribution of CoP and CuCoP (including different Cu doping contents) were investigated. The 3D charge density difference is shown in Figure 1b, in which the pure CoP charge is uniformly distributed, and the charge transfer increases obviously with the increase of the electron‐donor Cu content. Unbalanced charge density forms more chemical bonds with polysulfides, which further enhances the adsorption energy (−5.58 eV). Compared with pure CoP (−2.43 eV) with uniform charge distribution, this change is beneficial to enhance the binding energy with Li_2_S_6_. Simultaneously, electron‐donor Cu doping can induce more defects and vacancies of CoP, thus exposing more active sites for the polysulfides conversion and Li_2_S redox process (Li_2_S_4_ → Li_2_S and Li_2_S → Li_2_S_4_), thereby facilitating enhancing the Li_2_S redox kinetics and avoiding the accumulation of polysulfides/Li_2_S (inhibition of shuttle effect). Meanwhile, the 2D section of charge density clearly demonstrates that Cu atoms are in a state of charge depletion, while Co and P atoms near the Cu atoms are in a state of charge accumulation (Figure S6, Supporting Information). These results show that electron‐donor Cu doping redistributes the charge density of CoP, and the strongly electronegative Co^3+^ in CoP tended to seize the extra‐nuclear electrons of Cu atoms and converted into weakly electronegative Co^2+^ (Figure 1c). Therefore, the bond energy of the Co—S bonds formed between polysulfides and CoP catalyst is weakened (the length of the Co—S bonds are lengthened from 2.150 to 2.169 Å, Figure 1c), which is beneficial to the transfer of polysulfides and the Li_2_S diffusion and decomposition (Li_2_S → Li_2_S*_n_*) on CoP surface. The activation energy (*E*_a_) was calculated via Arrhenius equation, which is to further confirm that the electron‐donor Cu doping can effectively reduce the diffusion barrier and activation energy (Figure 1d,e).^[^ ^24^ ^]^ The conversion and adsorption mechanism of Li_2_S/polysulfides realized by the electron‐donor Cu‐doped CoP/MXene is depicted based on the DFT calculation, kinetics, and thermodynamics, as schematically illustrated in Figure 1f. In brief, electron‐donor Cu doping effectively improves the adsorption ability and catalytic activity of CoP/MXene, which ensures the orderly conversion of Li_2_S_8_ → Li_2_S_4_ → Li_2_S → Li_2_S*_n_* at a high‐sulfur‐loaded cathode.

### 2.2 Microstructures and Morphologies of Cu*_x_* Co_1−_ *_x_* P/MXene Electrocatalysts

Figure 3 shows the microstructures, morphologies, crystalline structures, and chemical environment on the surface of Cu*_x_* Co_1−_ *_x_* P/MXene and MXene, which were characterized through transmission electron microscopy (TEM) and scanning electron microscopy (SEM). Figure 3a and Figure S1b, Supporting Information, show a few‐layer Ti_3_C_2_‐MXene without stacking and curling, which is conducive to the in situ growth of Cu*_x_* Co_1−_ *_x_* P nanosheets. SEM images show that the Cu*_x_* Co_1−_ *_x_* P nanosheets are equably distributed on the surface of MXene, and different‐content Cu doping do not affect the microstructures and morphologies (Figure 3b–d and Figure S3, Supporting Information). Simultaneously, this uniformly grown 3D structure possesses high specific surface area (BET areal of 244 m^2^ g^−1^) and metalloid conductivity (Figures S4 and S22, Supporting Information). Figure 3e presents the high‐resolution TEM image of Cu_0.1_Co_0.9_P/MXene, on which two lattice planes with the fringe spacing of 0.23 and 0.19 nm are observed. And the two lattice planes belong to the (006) plane (Ti_3_C_2_‐MXene) and the (211) plane (Cu_0.1_Co_0.9_P), respectively (Figure 3e1,e_2_). Additionally, an obvious hetero‐interface between the two lattice planes can be observed, indicating that Cu_0.1_Co_0.9_P nanosheets are in situ grown on MXene (Figure 3e_3_). In addition, the defects induced by electron‐donor Cu doping are clearly observed in Figure 3e_4_ and Figure S7, Supporting Information, demonstrating the defect‐rich characters of Cu_0.1_Co_0.9_P/MXene. Energy‐dispersive X‐ray spectrum (Figure 3f and Figure S1d, Supporting Information) analysis verifies the uniform distribution of Ti, C, Co, P, and Cu elements on the entire Cu_0.1_Co_0.9_P/MXene.

### 2.3 Structural and Component Analysis of Cu*_x_* Co_1−_ *_x_* P/MXene Electrocatalysts

To further verify the formation of the Cu*_x_* Co_1−_ *_x_* P/MXene electrocatalysts, the detailed structural and component analysis was carried out by X‐ray diffraction (XRD) and X‐ray photoelectron spectroscopy (XPS). The XRD peaks of Cu*_x_* Co_1−_ *_x_* P/MXene and pure CoP/MXene in Figure 3g well matched with the standard XRD peaks of CoP (PDF#29‐0497), indicating the formation of CoP on MXene. It is noted that the XRD peaks shift to a lower angle compared with pure CoP/MXene. To observe the angle deviation intuitively, the area near the main peak (211 crystal plane) of Cu*_x_* Co_1−_ *_x_* P/MXene was scanned narrowly. As shown in Figure 3h, the peak of Cu*_x_* Co_1−_ *_x_* P/MXene have obvious deviation, and it is similar to the peak of the CuCo‐LDH/MXene in Figure S2, Supporting Information, which indicates that electron‐donor Cu doping leads to the change of (211) lattice plane, defects might occur simultaneously, and these variations possibly affect the number of active sites and catalytic efficiency, which is in favor of catalytic polysulfides/Li_2_S conversion with high efficiency.^[^ ^46^ ^]^ Furthermore, the XPS spectra in Figure 3i exhibits the survey scan of the Cu*_x_* Co_1−_ *_x_* P/MXene and pure CoP/MXene, the evident Cu 2p peak manifests the successful synthesis of Cu‐doped CoP/MXene.

The high‐resolution Co 2p XPS spectra in Cu*_x_* Co_1−_ *_x_* P/MXene (Figure S8a, Supporting Information) reveals seven separate peaks with Co—P, Co^3+^ (2p_3/2_ and 2p_1/3_), Co^2+^ (2p_3/2_ and 2p_1/3_), and accompanied by two satellite peaks (the satellite peak of Co 2p_3/2_ and Co 2p_1/2_ are denoted as Sat.).^[^ ^47^, ^48^ ^]^ By fitting the peak areas of Co^2+^ and Co^3+^, respectively, it can be clearly found that the proportion of the peak area of Co^2+^ (the content of Co^2+^) in Co 2p_3/2_ increases with the increase of electron‐donor Cu doping content (see Figure 3j and Table S1, Supporting Information). This may be due to the fact that the extra‐nuclear electrons of Cu atoms are captured by strong electronegativity Co^3+^ and converted into Co^2+^, which can also be verified by the charge density of Cu*_x_* Co_1−_ *_x_* P (Figure 1b). With the augment of electron‐donor Cu doping content, the charge transfer regions centered on Cu atoms also increase, and more Co^3+^ electrons are converted into Co^2+^. Compared with the Co 2p peaks of pure CoP/MXene, it could be found that the peaks of Co 2p in Cu*_x_* Co_1−_ *_x_* P/MXene has a weak shift (Figure S8a, Supporting Information); this change is attributed to the electronic interaction between Cu and Co atoms.^[^ ^49^ ^]^

The P 2p XPS spectra in Figure S8b, Supporting Information, could be deconvoluted into the three featured peaks of P‐O, P 2p_1/2_, and P 2p_3/2_. After electron‐donor Cu doping, the P 2p_1/2_ and P 2p_3/2_ peaks both shift to a lower field compared with those in CoP/MXene (Figure 3k), which may be due to P gaining electrons (i.e., unpaired electrons are formed due to defects). This is consistent with the above conclusion, that is, the P atoms are in a state of charge accumulation (Figure S6, Supporting Information). Besides, according to the variation of the P element states and the electronic structure for Cu*_x_* Co_1−_ *_x_* P/MXene, it can be explained that the defects induced by Cu doping leads to the formation of vacancies.

Thereby electron paramagnetic resonance (EPR) measurement was used to further verify the existence of vacancies in Cu*_x_* Co_1−_ *_x_* P/MXene. As shown in Figure 3l, the EPR spectra of Cu*_x_* Co_1−_ *_x_* P/MXene (*x* = 0.05, 0.1, and 0.15) electrocatalyst show strong signals, in which Cu_0.1_Co_0.9_P/MXene exhibits the strongest signal peak with a *g* value of 2.0046, but for CoP/MXene, there is no signals at the position. The results indicate that there are unpaired electrons in Cu*_x_* Co_1−_ *_x_* P/MXene, which could be attributed to that the electron‐donor Cu doping facilitated the formation of lattice vacancies in the CoP/MXene. Compared to CoP/MXene, Cu_0.05_Co_0.95_P/MXene, and Cu_0.15_Co_0.85_P/MXene, Cu_0.1_Co_0.9_P/MXene exhibits the strongest EPR signal, which validates the highest concentration of vacancy.^[^ ^50^, ^51^ ^]^ While, the signal intensity of Cu_0.15_Co_0.85_P/MXene is weaker than that of Cu_0.1_Co_0.9_P/MXene, which may be due to the defect recombination (annihilation) by the increase of Cu content.^[^ ^30^, ^52^ ^]^ These vacancies (point defect) may cause the charge transfer between surrounding atoms (regulating the electron density close to the Fermi level), optimize the electronic configuration and adsorption behaviors, and further increase the active centers, which are conducive to improve the overall catalytic activity of Cu*_x_* Co_1−_ *_x_* P/MXene.^[^ ^53^, ^54^ ^]^

### 2.4 Catalytic Kinetic and Thermodynamic Mechanism of Cu*_x_* Co_1−_ *_x_* P/MXene Electrocatalysts for Polysulfides and Li_2_S Redox

**Figure 4:**
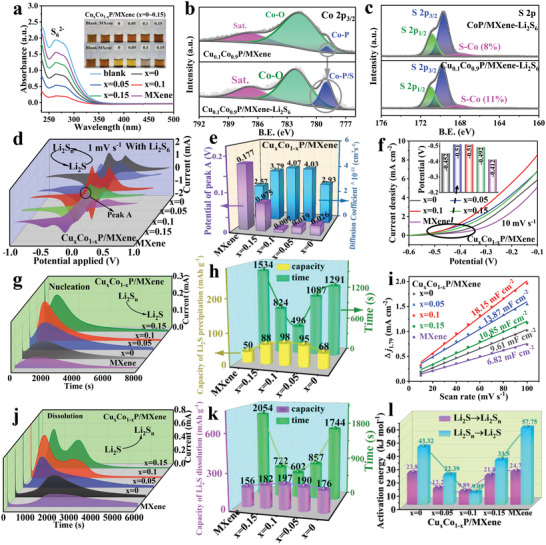
Catalytic properties and activation energy of Cu*_x_* Co_1−_ *_x_* P/MXene in comparison to CoP/MXene and MXene. a) UV–vis spectra of Li_2_S_6_ solution and the optical image (inset) after interaction. XPS spectra of b) Co 2p_3/2_ and c) S 2p in Cu_0.1_Co_0.9_P/MXene, Cu_0.1_Co_0.9_P/MXene‐Li_2_S_6_, CoP/MXene, and CoP/MXene‐Li_2_S_6_. d) CV profiles of symmetric cells using Li_2_S_6_‐containing electrolyte at the scan rate of 1 mV s^−1^. e) Comparison of peak potential and diffusion coefficient of Li^+^. f) LSV curves. g) Potentiostatic discharge and j) charge curves at 2.11 and 2.35 V, respectively. h) Precipitation and k) dissolution capacity and time of Li_2_S. i) Plots of current density difference (∆*j*) against scan rates of the five electrodes at 1.79 V. l) *E*_a_ of Li_2_S and Li_2_S*_n_* interconversion.

The prerequisite for the catalyst to function (e.g., inhibit polysulfides dissolution, promote redox kinetics of Li_2_S, and catalyze polysulfides conversion) is that it could effectively capture polysulfides during electrochemical conversion of LSB. Therefore, the adsorption ability of the Cu*_x_* Co_1−_ *_x_* P/MXene and MXene for Li_2_S_6_ was first tested (**Figure** **4**a). With the decrease of the concentration of Li_2_S*_n_* in the electrolyte, the color will change from brown to yellow, and gradually fade until it becomes colorless. The obvious difference in the color of Li_2_S_6_ solution indicates the different adsorption ability of Cu*_x_* Co_1−_ *_x_* P/MXene and MXene. Compared with MXene and Cu*_x_* Co_1−_ *_x_* P/MXene (*x* = 0, 0.05, and 0.1), the Li_2_S_6_ solution of Cu_0.1_Co_0.9_P/MXene is almost colorless, suggesting that Cu_0.1_Co_0.9_P/MXene has the strongest adsorption capacity and the fastest adsorption velocity for Li_2_S_6_. The weaker peak (260 nm) assigned to Li_2_S_6_ (Figure 4a) and the washed‐out solution in inset show that the adsorption ability for polysulfides, can be effectively boosted with the appropriate electron‐donor Cu doping (e.g., 10 at%).

To further explore the interaction between the Cu*_x_* Co_1−_ *_x_* P/MXene electrocatalyst and Li_2_S_6_, the surface chemical properties of Cu_0.1_Co_0.9_P/MXene and CoP/MXene before and after interacting with Li_2_S_6_ were analyzed by XPS spectra (Figure 4b,c). Co 2p_3/2_ XPS spectra of Cu_0.1_Co_0.9_P/MXene, Cu_0.1_Co_0.9_P/MXene‐Li_2_S_6_, CoP/MXene, and CoP/MXene‐Li_2_S_6_ in Figure 4b and Figure S9a, Supporting Information, show that there are three peaks assigned to Co—P bond (779.05 eV for Cu_0.1_Co_0.9_P/MXene, 779.0 eV for Cu_0.1_Co_0.9_P/MXene‐Li_2_S_6_), Co—O (781.85 eV for Cu_0.1_Co_0.9_P/MXene, 782.2 eV for Cu_0.1_Co_0.9_P/MXene‐Li_2_S_6_), and the satellite peak (786.9 eV for Cu_0.1_Co_0.9_P/MXene, 786.75 eV for Cu_0.1_Co_0.9_P/MXene‐Li_2_S_6_), respectively. The relative intensity of Co—P/S peak are stronger than Co—P peak in Cu_0.1_Co_0.9_P/MXene‐Li_2_S_6_ and CoP/MXene‐Li_2_S_6_, which shows more obvious intensity variation in Cu_0.1_Co_0.9_P/MXene‐Li_2_S_6_ Co2p_3/2_ XPS spectra. The stronger peak of Co—P/S is attributed to the formation of Co—S bonds (between Co atoms in Cu_0.1_Co_0.9_P/MXene and CoP/MXene and S atoms in polysulfides), indicating that Cu_0.1_Co_0.9_P/MXene anchors Li_2_S_6_ with more Co—S bonds, which may be assigned to the existence of more vacancies and defect sites in Cu_0.1_Co_0.9_P/MXene. Figure 4c exhibits the high‐resolution S 2p spectra of Cu_0.1_Co_0.9_P/MXene‐Li_2_S_6_ and CoP/MXene‐Li_2_S_6_, which display three peaks at 168.05, 169.85, and 171.05 eV, and 167.7, 169.65, and 170.85 eV, corresponding to S—Co bond, S 2p_3/2_, and S 2p_1/2_, respectively. Compared with the S—Co peak of CoP/MXene‐Li_2_S_6_ (8%), the relative intensity of Cu_0.1_Co_0.9_P/MXene‐Li_2_S_6_ (11%) is higher. This is consistent with Co—P/S peak of the Co 2p_3/2_ spectra in CoP/MXene‐Li_2_S_6_ and Cu_0.1_Co_0.9_P/MXene‐Li_2_S_6_, which can be assigned to that the defects induced by electron‐donor Cu doping are more likely to chemically interact with polysulfides. This further verifies that Cu doping makes CoP/MXene has stronger adsorption ability toward Li_2_S_6_. Furthermore, after the interaction of Co atoms with S atoms, the peaks of Co—P shift to lower binding energies, which manifests the electrons transfer from Li_2_S*_n_* to CoP surface. Simultaneously, the Ti 2p XPS spectra of Cu_0.1_Co_0.9_P/MXene after interacting with Li_2_S_6_ exhibits a new set of peaks (Ti–S) (Figure S9b, Supporting Information), which suggests that Ti_3_C_2_‐MXene can also anchor Li_2_S*_n_* through chemical bonds. The above results show that Cu*_x_* Co_1−_ *_x_* P/MXene electrocatalyst has a good adsorption ability toward Li_2_S_6_.^[^ ^55^ ^]^

To explore the catalytic effects of Cu*_x_* Co_1−_ *_x_* P/MXene electrocatalyst, the redox potentials in cyclic voltammetry (CV) curves under various rates of symmetric cells were evaluated (Figure 4d and Figure S10, Supporting Information). The clearly separated redox peaks in Figure 4d and Figure S11, Supporting Information, can be attributed to the stepwise conversion of Li_2_S*_n_*. The cathodic scan and anodic scan represent the electrochemical conversion process of Li_2_S*_n_* being reduced to Li_2_S and Li_2_S being oxidized to Li_2_S*_n_*, respectively. Compared with MXene, the clearly separated double peaks of CoP/MXene represent the rapid redox conversion of Li_2_S*_n_*. It is noted that the CV curve of Cu_0.1_Co_0.9_P/MXene proves three pairs of redox peaks and the highest current response, indicating the fastest redox kinetics of Li_2_S*_n_*.^[^ ^56^, ^57^, ^58^, ^59^ ^]^ Inversely, the cell with MXene exhibits inconspicuous double peaks, a low current response, and large voltage hysteresis (Figure 4e).

Concurrently, as the scan rate increases, the profile of CV curves remains consistent, indicating that Cu_0.1_Co_0.9_P/MXene has significantly better catalytic performance and favorable durability than the comparative materials (Figure S10c, Supporting Information). Figure S10f, Supporting Information, shows the relation of peak A response current with scan rates, where the ion diffusion coefficient (*D*_Li+_) was calculated according to the Randles–Sevcik equation.^[^ ^60^, ^61^ ^]^ According to Figure S10f, Supporting Information, and Figure 4e, the *D*_Li+_ values for Cu*_x_* Co_1−_ *_x_* P/MXene and MXene at peak A are 2.93 × 10^−11^, 4.03 × 10^−11^, 4.07 × 10^−11^, 3.79 × 10^−11^, and 2.57 × 10^−11^ cm^−2^ s^−1^, respectively. This result pithily confirms that Cu doping accelerates the diffusion rate of Li^+^, and the effect is the best when the content of Cu doping is 10 at%. The catalytic activity of Cu*_x_* Co_1−_ *_x_* P/MXene and MXene on polysulfides conversion was further investigated by linear sweep voltammetry (LSV) (Figure 4f). The LSV curves clearly shows that the Cu_0.1_Co_0.9_P/MXene electrode (red curve) has the lowest onset potential near −0.51 V and the highest oxidation current, indicating that Cu_0.1_Co_0.9_P/MXene greatly promotes the conversion kinetics from Li_2_S to polysulfides.

To further demonstrate the bidirectional catalytic effect of Cu_0.1_Co_0.9_P/MXene electrocatalyst for polysulfide, the redox kinetics of Li_2_S were studied through Li_2_S nucleation and dissolution experiments.^[^ ^62^, ^63^, ^64^ ^]^ The nucleation process (Figure 4g and Figure S12, Supporting Information) and dissolution process (Figure 4j and Figure S13, Supporting Information) of Li_2_S represent the conversion of polysulfides into Li_2_S and Li_2_S into polysulfides, respectively. The time for occurred current peak in potentiostatic discharging curves demonstrate that the Cu_0.1_Co_0.9_P/MXene electrode significantly accelerates the Li_2_S nucleation (≈496 s) and dissolution (≈602 s), compared with those of CoP/MXene (≈1291 s, 1744 s), Cu_0.05_Co_0.95_P/MXene (≈1087 s, 857 s), Cu_0.15_Co_0.85_P/MXene (≈824 s, 722 s), and MXene electrodes (≈1534 s, 2054 s) (Figure 4h,k). Furthermore, the Li_2_S nucleation and dissolution capacity on Cu*_x_* Co_1−_ *_x_* P/MXene and MXene surfaces were gained through the Faraday's law. The analysis and calculation results in Figure 4h,k show that Cu_0.1_Co_0.9_P/MXene electrode exhibits the highest nucleation capacity (98 mAh g^−1^) and dissolution capacity (197 mAh g^−1^) of Li_2_S than those of CoP/MXene (68 and 176 mAh g^−1^), Cu_0.05_Co_0.95_P/MXene (95 and 190 mAh g^−1^), Cu_0.15_Co_0.85_P/MXene (88 and 182 mAh g^−1^), and MXene electrodes (50 and 156 mAh g^−1^). These data manifest that the Cu_0.1_Co_0.9_P/MXene electrode has the highest efficiency catalytic ability for Li_2_S*_n_* to Li_2_S and Li_2_S to Li_2_S*_n_* conversion, which can greatly shorten the conversion reaction time, reduce the accumulation of polysulfides, and accelerate the Li_2_S redox kinetics.

The different performance of Cu*_x_* Co_1−_ *_x_* P/MXene catalysts in terms of catalysis is possibly because Cu‐doping can induce more defects and vacancies to CoP/MXene, thus affecting the anchoring ability (capture polysulfides) and catalytic activity (Li_2_S*_n_* conversion). Hence, the electrochemical active surface area (ECSA) was calculated by the electrochemical capacity of the Cu*_x_* Co_1−_ *_x_* P/MXene in the diluted Li_2_S_6_ solution, which could be gauged by CV curves under multifarious rates in non‐Faradaic potential region (Figure S14, Supporting Information). Figure 4i shows the currents against the scan rates, the slope of curve represents the double layer capacitance (*C*_dl_) of Cu*_x_* Co_1−_ *_x_* P/MXene electrode, and its value can directly reflect ECSA of electrode. Cu_0.1_Co_0.9_P/MXene displays the highest *C*_dl_ value (18.15 mF cm^−2^) than those of Cu_0.05_Co_0.95_P/MXene (13.87 mF cm^−2^), Cu_0.15_Co_0.85_P/MXene (10.85 mF cm^−2^), CoP/MXene (9.61 mF cm^−2^), and MXene (6.82 mF cm^−2^), manifesting that electron‐donor Cu doping could expose more active sites, and the Cu doping content with 10% is the optimal doping level (Figure S14f, Supporting Information).

To further decipher the catalytic mechanism of polysulfides on Cu*_x_* Co_1−_ *_x_* P/MXene and MXene surface, the activation energies (*E*_a_) of the bidirectional conversion between Li_2_S*_n_* and Li_2_S were estimated by the Arrhenius equation.^[^ ^24^ ^]^ To obtain the connection between a peak current and temperature, the CV curves of Cu*_x_* Co_1−_ *_x_* P/MXene and MXene electrodes at different temperatures were tested (Figure S15, Supporting Information), respectively. In contrast, the CV curves of Cu*_x_* Co_1−_ *_x_* P/MXene (*x* = 0.05 0.1, and 0.15) electrodes exhibit smaller peak width at half‐height than those of CoP/MXene and MXene, implying more rapid catalytic kinetics. The two peaks of cathodic scans correspond to the two conversion processes of Li_2_S_8_ → Li_2_S_6_ (near 2.25 V) and Li_2_S_4_ → Li_2_S (near 2.08 V), respectively. Considering the contribution capacity of each peak, the peaks around 2.41 (peak A) and 2.08 V (peak C) were selected to calculate the activation energy of the Li_2_S redox process. Figure S15f, Supporting Information, shows the fitting lines of the peak current and temperature for the five hosts. Peaks A and C represent the conversion process of Li_2_S → Li_2_S*_n_* and Li_2_S_4_ → Li_2_S, respectively. Figure 4l shows that Cu_0.1_Co_0.9_P/MXene exhibits the lowest energy barrier of Li_2_S_4_ → Li_2_S and Li_2_S → Li_2_S*_n_* (9.05 and 9.89 kJ mol^−1^) than those of CoP/MXene (43.32 and 23.8 kJ mol^−1^), Cu_0.05_Co_0.95_P/MXene (22.39 and 12.2 kJ mol^−1^), Cu_0.15_Co_0.85_P/MXene (33.5 and 21.8 kJ mol^−1^), and MXene (57.75 and 24.7 kJ mol^−1^). The result proves that Cu*_x_* Co_1−_ *_x_* P/MXene effectively reduces the *E*_a_ for Li_2_S nucleation and dissolution compared to pure MXene and CoP/MXene, thereby accelerating the Li_2_S redox kinetics, and improving the nucleation and dissolution capacity. Simultaneously, the above analysis can decipher that the remarkable catalytic activity of Cu_0.1_Co_0.9_P/MXene, which is attribute to that electron‐donor Cu doping exposes more active sites (induced the formation of vacancies), enhances the adsorption capacity for polysulfides, reduces the energy barrier of electrochemical reactions, and accelerates the reaction kinetics.

### 2.5 Density Functional Theory Calculation Simulation Analysis

**Figure 5:**
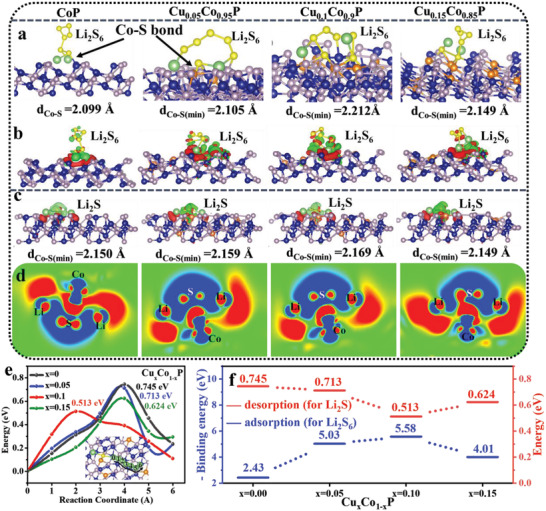
Theoretical calculations for reaction mechanism. a) Side view of the optimized model after the Cu*_x_* Co_1−_ *_x_* P adsorbs Li_2_S_6_. The charge density of b) Cu*_x_* Co_1−_ *_x_* P‐Li_2_S_6_ and c) Cu*_x_* Co_1−_ *_x_* P‐Li_2_S 3D isosurfaces and d) 2D slices (isosurface level is set to 0.004 e Å^−3^) (red and blue/green represent the electron accumulate and depletion regions, respectively). e) Top view of the Li_2_S diffusion path on the Cu_0.1_Co_0.9_P surface (insert) and the corresponding energy barrier. f) The binding energy of Li_2_S_6_ and the desorption energy barrier of Li_2_S on the Cu*_x_* Co_1−_ *_x_* P surface.

To further understand the adsorption ability toward Li_2_S*_n_* and the Li_2_S decomposition energy barrier on the Cu*_x_* Co_1−_ *_x_* P/MXene surface, DFT was used to simulate the interaction between Li_2_S_6_ and the catalyst and the decomposition path of Li_2_S. The CoP nanosheets in Cu*_x_* Co_1−_ *_x_* P/MXene composites play a decisive role in the catalytic conversion of Li_2_S*_n_* and Li_2_S. Therefore, the initial structure modules of Cu*_x_* Co_1−_ *_x_* P (*x* = 0, 0.05, 0.1, and 0.15) were constructed, as shown in Figure S16, Supporting Information (MXene mainly serves as the conductive matrix for the growth of CoP). There are different numbers of Co atoms replaced by Cu atoms, representing CoP with different Cu‐doping amounts. **Figure** **5**a and Figure S17, Supporting Information, present the side view and top view of model after Cu*_x_* Co_1−_ *_x_* P (*x* = 0, 0.05, 0.1, and 0.15) absorbs Li_2_S_6_. The bond length was measured in the model, and the Co—S bonds length (2.212 Å) (choose the shortest from multiple Co—S bonds of Cu*_x_* Co_1−_ *_x_* P‐Li_2_S_6_) in Cu_0.1_Co_0.9_P‐Li_2_S_6_ were longer than that of CoP‐Li_2_S_6_ (2.099 Å), Cu_0.05_Co_0.95_P‐Li_2_S_6_ (2.105 Å), and Cu_0.15_Co_0.85_P‐Li_2_S_6_ (2.149 Å).

It is worth noting that there are different numbers of Co—S bonds in Cu*_x_* Co_1−_ *_x_* P‐Li_2_S_6_, for example, CoP‐Li_2_S_6_ only forms one Co—S bond, and the Cu*_x_* Co_1−_ *_x_* P‐Li_2_S_6_ (*x* = 0.05, 0.1, and 0.15) have at least two Co—S bonds (there is still a small amount of Cu—S bonds and Li—P bonds). This is consistent with the increase in Co—P/S peak area in Co 2p_3/2_ XPS spectra (Figure 4b), which indicates that electron donor Cu doping promotes the formation of more chemical bonds and thereby enhances the adsorption ability of Cu*_x_* Co_1−_ *_x_* P for polysulfides. Besides, the charge density of Cu*_x_* Co_1−_ *_x_* P‐Li_2_S_6_ (*x* = 0, 0.05, 0.1, and 0.15) exhibits obvious increase of charge transfer after electron donor Cu doping (Figure 5b), which also indicates that there was a strong interaction between Cu*_x_* Co_1−_ *_x_* P and Li_2_S_6_, and more Co and S atoms (it may also include P and Li atoms) participate in the bonding. Figure 5c and Figure S18, Supporting Information, show the charge density of Cu*_x_* Co_1−_ *_x_* P‐Li_2_S. There are two Co—S bonds with different lengths in the optimized model, and the shortest one was selected for comparison. Unsurprisingly, with the content of Cu‐doping increases, the length of the Co—S bond becomes longer (Cu*_x_* Co_1−_ *_x_* P‐Li_2_S, *x* = 0, 0.05, and 0.1 correspond to 2.150, 2.159, and 2.169 Å, respectively). However, this trend is broken because the length of Co—S bond in Cu_0.15_Co_0.85_P‐Li_2_S is shorter than that in Cu_0.1_Co_0.9_P‐Li_2_S when the Cu‐doping content reaches 15 at%. The variation trend of the influence of Cu doping content on the length of Co—S bond is also reflected in Cu*_x_* Co_1−_ *_x_* P‐Li_2_S_6_, which may be attributed to the increased of Co^2+^ with weaker electronegativity than that of Co^3+^ in Cu*_x_* Co_1−_ *_x_* P/MXene (Figure 3j), while the shortening of Co—S bond in Cu_0.15_Co_0.85_P‐Li_2_S_6_/Li_2_S may be related to the formation of Cu—S bonds. Ulteriorly, the charge around the Co and S atoms depletes, while the charge accumulates between Co and S atoms, which suggests that the Co—S bond between the Co and S atoms is built (Figure 5d). By contrast, the charge density between CoP and Li_2_S is greater than that of Cu*_x_* Co_1−_ *_x_* P (*x* = 0.05, 0.1, and 0.15), which results in a more stable binding energy (i.e., a higher decomposition energy barrier). To further understand the relationship between the energy of Co—S bonds and the content of Cu doping, the diffusion path corresponds to the decomposition energy barrier for Li_2_S and the binding energy for Li_2_S_6_ on the surface of Cu*_x_* Co_1−_ *_x_* P were evaluated using DFT calculations (Figure 5e and Figure S19, Supporting Information) (as the diffusion path is the same as Li_2_S, the insert only shows the Cu_0.1_Co_0.9_P‐Li_2_S as the representative).

The Li_2_S on Cu_0.1_Co_0.9_P surface exhibits a diffusion energy barrier of 0.513 eV, lower than those on CoP surface (0.745 eV), Cu_0.05_Co_0.95_P surface (0.713 eV), and Cu_0.15_Co_0.85_P surface (0.624 eV) (Figure 5f). Simultaneously, the binding energy with Cu*_x_* Co_1−_ *_x_* P and Li_2_S_6_ are shown Figure 5f. The lowest energy barrier and highest binding energy (5.58 eV) of Cu_0.1_Co_0.9_P may be attributed to the formation of the longer Co—S bonds in Cu_0.1_Co_0.9_P/MXene‐Li_2_S and the more Co—S bonds in Cu_0.1_Co_0.9_P/MXene‐Li_2_S_6_. Low diffusion barrier for Li_2_S and high binding energy for polysulfides are beneficial to the detachment of Li_2_S from Cu_0.1_Co_0.9_P/MXene surface during oxidation and the capture of Li_2_S_6_ by Cu_0.1_Co_0.9_P/MXene during reduction. The DFT calculation results are completely consistent with the results of the above static adsorption experiments, kinetic experiments, and thermodynamics (activation energy calculations), indicating that electron‐donor Cu doping (especially the Cu doping with 10 at%) plays an important role in improving the catalytic performance of CoP/MXene. Because of these advantageous features, an effective suppression on polysulfides shuttling would be demonstrated when Cu*_x_* Co_1−_ *_x_* P/MXene was adopted as the sulfur host.

### 2.6 Comparative Electrochemical Performance of Cu*_x_* Co_1−_ *_x_* P/MXene Sulfur Composite Cathodes at a Routine Electrolyte

**Figure 6:**
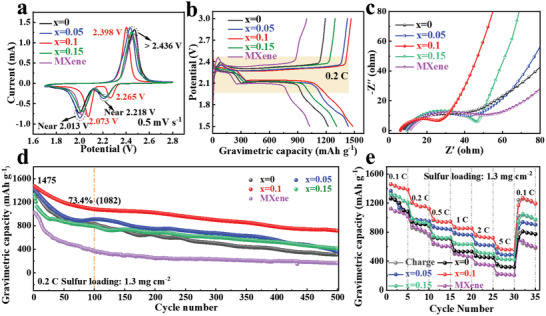
Electrochemical performance of Cu*_x_* Co_1−_ *_x_* P/MXene and MXene sulfur cathodes in LSBs. a) CV curves at 0.5 mV s^−1^. b) GDC profiles at 0.2 C. c) EIS spectra. d) Cycling stability tests at 0.2 C. e) Rate performance.

As Cu*_x_* Co_1−_ *_x_* P/MXene owns an excellent conductivity, a fast adsorption ability for polysulfides, and fast kinetics and low *E*_a_ for Li_2_S redox, the electrochemical performance of LSBs using Cu*_x_* Co_1−_ *_x_* P/MXene as sulfur hosts with the comparison to pure CoP/MXene and MXene were evaluated, respectively. The S/Cu*_x_* Co_1−_ *_x_* P/MXene composites (sulfur content ≈ 78.6 wt%) (Detected by thermogravimetric analysis, Figure S20a, Supporting Information) were prepared through a melt‐infusion strategy. **Figure** **6**a shows the CV profiles of S/Cu*_x_* Co_1−_ *_x_* P/MXene and S/MXene cathodes at 0.5 mV s^−1^. The S/Cu_0.1_Co_0.9_P/MXene shows obvious redox peaks at 2.265 V (corresponding to Li_2_S_8_ → Li_2_S*_n_*), 2.073 V (Li_2_S*_n_* → Li_2_S), and 2.398 V (Li_2_S_2_/Li_2_S to Li_2_S_8_), respectively. Compared with S/Cu_0.1_Co_0.9_P/MXene, the oxidation peak and two reduction peaks for S/CoP/MXene, S/Cu_0.05_Co_0.95_P/MXene, S/Cu_0.15_Co_0.85_P/MXene, and S/MXene exhibit a shift to the right for the anodic peak (≈2.436 V) and to the left for the cathode peaks (≈2.218 and ≈2.013 V), respectively. The narrowest half‐peak width and the smallest polarization suggest the fastest redox kinetics of S/Cu_0.1_Co_0.9_P/MXene, which is beneficial to the polysulfides conversion.

Figure 6b presents the galvanostatic discharge/charge (GDC) curves of S/Cu*_x_* Co_1−_ *_x_* P/MXene and S/MXene. The initial gravimetric capacity of S/Cu_0.1_Co_0.9_P/MXene cathode is 1475 mAh g^−1^ at 0.2 C, which is higher than those of S/Cu_0.05_Co_0.95_P/MXene (1431 mAh g^−1^), S/Cu_0.15_Co_0.85_P/MXene (1313 mAh g^−1^), S/CoP/MXene (1223 mAh g^−1^), and S/MXene cathodes (1025 mAh g^−1^). The high gravimetric capacity of S/Cu_0.1_Co_0.9_P/MXene is attributed to the platform at ≈2.11 V contributing to a large capacity, indicating that Cu_0.1_Co_0.9_P/MXene promotes the efficient conversion toward polysulfides. Electrochemical impedance spectrum (EIS) of S/Cu_0.1_Co_0.9_P/MXene exhibited a smaller semicircle diameter (Figure 6c), which indicates a faster charge transfer.

Figure 6d presents the comparative long cycle stability with 500 cycles of Cu*_x_* Co_1−_ *_x_* P/MXene and MXene sulfur cathodes at 0.2 C. S/Cu_0.1_Co_0.9_P/MXene shows high initial gravimetric capacities of 1475 mAh g^−1^, and it decays to 1082, 1020, and 716 mAh g^−1^ at 100, 200, and 500 cycles, the corresponding capacity retention is 73.4%, 69.2%, and 48.6%, respectively. For contrast, S/Cu*_x_* Co_1−_ *_x_* P/MXene (*x* = 0, 0.05, and 0.15) and S/MXene attenuate to ≈57.6%, 63.7%, 59.6%, and 28.5% after 100 cycles, respectively. The excellent capacity retention rate of S/Cu_0.1_Co_0.9_P/MXene implies a superior catalytic ability for polysulfide redox conversion and a good cycle stability for inhibiting polysulfide shuttling.

Figure 6e presents the comparative rate capability of S/Cu*_x_* Co_1−_ *_x_* P/MXene and S/MXene cathodes. The Cu_0.1_Co_0.9_P/MXene cathode demonstrates much higher specific capacities of 1463, 1212, 976, 872, 763, and 599 mAh g^−1^ than those of S/Cu*_x_* Co_1−_ *_x_* P/MXene (*x* = 0, 0.05, and 0.15) and S/MXene at 0.1, 0.2, 0.5, 1, 2, and 5 C, respectively. It is noted that the charge/discharge profiles (Figure S21, Supporting Information) of S/Cu_0.1_Co_0.9_P/MXene exhibits the smallest polarization (202 mV) and the highest specific capacity (879 mAh g^−1^) than those of S/CoP/MXene (242 mV and 540 mAh g^−1^), S/Cu_0.05_Co_0.95_P/MXene (221 mV and 785 mAh g^−1^), S/Cu_0.15_Co_0.85_P/MXene (232 mV and 667 mAh g^−1^), and S/MXene cathodes (305 mV and 511 mAh g^−1^) at 1 C. The above results show that electron donor Cu doping (especially the doping content 10 at%) can accelerate redox kinetics and enhance the utilization of S and polysulfides. The excellent rate performance and capacity recovery performance (1271 mAh g^−1^) for S/Cu_0.1_Co_0.9_P/MXene indicate the high‐efficiency catalytic ability for Li_2_S redox conversion, which could be attributed to that the electron‐donor Cu doping improves efficiently the intrinsically catalytic ability and reduces the *E*_a_ of the Li_2_S redox reaction. Therefore, the Cu_0.1_Co_0.9_P/MXene is chosen as the representative for further research.

### 2.7 Fabrication and Characterization of Dense S/Cu_0.1_Co_0.9_P/MXene Monolith

Despite the optimal Cu_0.1_Co_0.9_P/MXene as an efficient sulfur host showing a high gravimetric performance, the volumetric performance is also a key factor for the LSBs commercialization. For enhancing the volumetric performance of LSBs, the as‐fabricated S/Cu_0.1_Co_0.9_P/MXene was first assembled into a hydrogel via using a low content of GO (≈15 wt%) as the “assembling agent” under the mixed solvothermal of 180 °C for 20 h, and then, a highly dense S/Cu_0.1_Co_0.9_P/MXene monolith was acquired by drying the S/Cu_0.1_Co_0.9_P/MXene hydrogel with 30 °C (Figure 2a, its specific fabrication was given in Experimental Section).

Figure 2a displays the volume change of S/Cu_0.1_Co_0.9_P/MXene hydrogel before and after air‐drying. Before drying, the volume of a cylindrical S/Cu_0.1_Co_0.9_P/MXene hydrogel is ≈2.32 cm^3^ (parent hydrogel). After air drying, its volume was sharply shrunk to merely ≈0.0577 cm^3^ with ≈1/40 of initial volume, and compact and dense microstructures without any macroporous structures (low BET surface area of 4.83 m^2^g^−1^) was presented (Figure 2b and Figure S22, Supporting Information), implying that abundant void and large pores can be compressed using our air‐drying strategy, so the compact, dense sulfur structure can be formed. And the apparent density of the dense sulfur monolith can reach 1.95 g cm^−3^ (the weight: ≈0.1125 g) (see Section S3.3, Supporting Information). Moreover, compared to S/Cu_0.1_Co_0.9_P/MXene composites, the XRD pattern of dense S/Cu_0.1_Co_0.9_P/MXene monolith also presented the characteristic peaks of original sulfur (Figure S23, Supporting Information), indicating that original sulfur was retained well though experiencing high‐temperature hydrothermal treatment. Furthermore, the accurate sulfur content (≈66.4 wt%) and uniform distribution in dense S/Cu_0.1_Co_0.9_P/MXene monolith are exhibited in Figure S20b, Supporting Information, and Figure 2b_1_–b_6_, respectively. The electrical conductivity of S/Cu_0.1_Co_0.9_P/MXene is 283 S m^−1^ (Figure 2c, see Section S3.4, Supporting Information). Moreover, the electrolyte wettability of dense S/Cu_0.1_Co_0.9_P/MXene monolith particles was elevated by using a contact angle experiment, as displayed in Figure 2d and Figure S24, Supporting Information. After electrolyte wettability, the contact angle is ≈10°, and the excessive electrolyte could still be quickly absorbed by dense S/Cu_0.1_Co_0.9_P/MXene monolith. This implies a good electrolyte wettability and permeability of dense S/Cu_0.1_Co_0.9_P/MXene monolith, which is beneficial to the electrolyte penetration into the inner of dense sulfur cathode for good ion transportation.

### 2.8 The Areal and Volumetric Capacities of Dense S/Cu_0.1_Co_0.9_P/MXene Cathode under Routine and Lean Electrolyte

Owing to the unique characteristics of compact structure with high density, high conductivity, and good electrolyte penetration, the dense S/Cu_0.1_Co_0.9_P/MXene monolith cathode could endow an excellent volumetric performance for LSBs. Because of the high conductivity (283 S m^−1^) of dense S/Cu_0.1_Co_0.9_P/MXene, no conductive additive was used to further enhance the sulfur content in the whole cathode. For elevating the volumetric capacity for the whole sulfur cathode, a compacted density of the dense S/Cu_0.1_Co_0.9_P/MXene cathode was calculated (1.93 g cm^−3^) (Figure S25, Supporting Information, its detailed calculation, see Section S3.3, Supporting Information) The initial volumetric capacity of the dense S/Cu_0.1_Co_0.9_P/MXene cathode at routine sulfur loading (1.3 mg cm^−2^) and electrolyte (15 µL mg_s_^−1^) is as high as 1664 Ah L^−1^ (1443 mAh g^−1^ based on S for gravimetric capacity) at 0.1 C (Herein, the volumetric capacity was calculated by the total volume of the whole cathode), and its volumetric capacity was retained at 1305 Ah L^−1^ with 82.6% capacity retention after 100 cycles 0.2 C (its corresponding GDC profiles are given in Figure S26, Supporting Information). The dense sulfur cathode was first activated at 0.1 C for 5 cycles and then tested under 0.2 C (1370 mAh g^−1^) (Figure 2e). These results demonstrate dense S/Cu_0.1_Co_0.9_P/MXene monolith cathode possessing a high initial capacity and well cycle stability.

In addition, a low‐dosage electrolyte plus a thick sulfur cathode is also indispensable for the realistic application of LSBs.^[^ ^65^ ^]^ Under a low‐dosage electrolyte of 5.0, even with 3.5 µL mg_s_^−1^ (Figure 2f,g), the electrochemical performance of dense S/Cu_0.1_Co_0.9_P/MXene cathode with a high sulfur content (≈5.1 mg cm^−2^) were also evaluated. In comparison to the routine electrolyte, the GCD curves of thick yet dense sulfur cathode at low‐dosage electrolyte (E/S ratios = 5.0 and 3.5 µL mg_s_^−1^) display small voltage polarizations and clear GCD plateaus (Figure 2f). And the further cycle stability with 100 cycles (0.1 C) also demonstrates the similar results with the good electrochemical performance maintenance under the low‐dosage electrolyte (Figure 2e). The thick yet dense S/Cu_0.1_Co_0.9_P/MXene cathode (S loading of 5.1 mg cm^−2^) displays the initial areal capacities of 6.36, 5.66, and 5.40 mAh cm^−2^ with the capacity retention of 76.3%, 79.9%, and 79.4% under the E/S ratios of 15.0, 5.0, and 3.5 µL mg_s_^−1^, respectively (Figure 2g). More surprisingly, the volumetric capacity of the dense thick S/Cu_0.1_Co_0.9_P/MXene monolith cathode still retained at 1280 or 1219 Ah L^−1^, and a good cycle stability with the capacity retention of 73.4% after 100 cycles and 79.4% after 50 cycles at low‐dosage electrolyte of 5.0 and 3.5 µL mg_s_^−1^ was presented, respectively (Figure 2e and Figure S27, Supporting Information). The results suggest that a high volumetric capacity was well maintained even at the low E/S of 3.5 µL mg_s_^−1^, and the volumetric capacity is higher than those advanced sulfur cathodes under the low‐dosage electrolyte (see Figure 2i and Table S2, Supporting Information). This further manifests a high sulfur utilization rate and suppressed polysulfide shuttling at the low‐dosage electrolyte. Besides, a thicker dense S/Cu_0.1_Co_0.9_P/MXene cathode (sulfur loading = 9.1 mg cm^−2^) give a larger areal capacity of 8.3 mAh cm^−2^ and a superior capacity retention of 76.99% (6.39 mAh cm^−2^) after 50 cycles at 0.1 C in the E/S of 5.0 µL mg_s_^−1^ (Figure 2h). These key factors for the practical LSBs including areal capacity, sulfur cathode density, E/S ratio, and volumetric capacity at an excellent level are displayed in Figure 2i and Table S2, Supporting Information. This indicates that it is of great significance and also provides a new strategy to integrate electron‐donor Cu‐doped CoP/MXene and dense‐sulfur engineering for acquiring high‐volumetric/areal‐capacity LSBs under low‐dosage electrolyte in realistic applications.

## 3 Conclusion

In summary, the Li_2_S redox kinetics were successfully manipulated by electron‐donor Cu doping, and the optimal Cu_0.1_Co_0.9_P/MXene bidirectional electrocatalyst as the efficient sulfur host gives the best gravimetric capacity and long lifespan. Through integrating the dense‐sulfur engineering, the dense S/CuCoP/MXene cathode (density = 1.95 g cm^−3^, conductivity = 283 S m^−1^) presents the ultrahigh volumetric capacity of 1664 Ah L^−1^ (based on the total volume of the sulfur cathode) at 0.1 C in a routine electrolyte. More impressively, under a low‐dosage electrolyte of 5.0 µL mg_s_^−1^, the dense yet thick sulfur cathode still retains a high volumetric capacity of 1280 Ah L^−1^ and a large areal capacity of ≈8.3 mAh cm^−2^ at 0.1 C. Besides, the manipulating mechanism of Li_2_S redox kinetics was verified by systematical analysis of DFT calculation, kinetics, and thermodynamics, which mainly originates from that the electron‐donor Cu doping can easily make the strongly electronegative Co^3+^ in CoP converted into weakly electronegative Co^2+^, thus lengthening the bond length of Co—S bond, further facilitating the diffusion of polysulfides and Li_2_S on the electrocatalyst surface, so the diffusion energy barrier and activation energy of Li_2_S nucleation and decomposition is reduced, thus intrinsically boosting the Li_2_S redox kinetics. Second, Cu doping can induce more defects and vacancies of CoP, thus exposing more active sites for the catalytic conversion of sulfur species, thereby facilitating to enhance the Li_2_S redox kinetics; Third, the dense sulfur structure can endow large volumetric performance, and the hydrophilic MXene and graphene can ensure the good electrolyte permeation in a low‐dosage electrolyte. By the above synergistic effect, the dense monolith cathode achieves the high volumetric and areal performance in low‐dosage electrolyte for LSBs. Therefore, this work deeply understands the manipulating mechanism for Li_2_S redox kinetics via electronic donor doping at the electronic and atomic level, and also provides the dependable design principle for high‐volumetric‐capacity LSBs in low‐dosage electrolyte through integrating a bidirectional electrocatalyst with manipulated Li_2_S redox kinetics and dense‐sulfur engineering.

## 4 Experimental Section

### Synthesis of Cu*_x_* Co_1−_ *_x_* P/MXene Catalyst

First, few‐layer Ti_3_C_2_‐MXene was synthesized by the reported methods.^[^ ^44^ ^]^ Next Cu*_x_* Co_1−_ *_x_* LDH/MXene (x = 0, 0.05, 0.1, and 0.15) was prepared by a simple hydrothermal process. For example, when *x* = 0.1, solution A: 0.9 mmol of Co(NO_3_)_2_·6H_2_O (Aladdin, 99.9%) and 0.1 mmol of Cu(NO_3_)_2_·3H_2_O (Aladdin, 99.9%) (The total number of moles of Co^2+^ and Cu^2+^ was 1.0 mmol) were added into 15 mL of ultrapure water with stirring for 30 min. And solution B: 30 mg of as‐prepared MXene was ultrasonically dispersed into 20 mL of ethylene glycol. Afterward, solution A was added into solution B under vigorous stirring, and the mixed solution was continuously stirred in nitrogen protection until a homogeneous solution was formed (more than 2 h). Then, the mixed solution was heated to 120 °C and kept for 6 h. After natural cooling, the precipitate was taken out and washed with ultrapure water for at least three times, and then was freeze‐dried to obtain Cu_0.1_Co_0.9_LDH/MXene. Finally, the Cu_0.1_Co_0.9_LDH/MXene and sodium hypophosphite (NaH_2_PO_2_, Aladdin, 99.9%) (the molar ratio was 1:10) were put into the tube furnace and heated to 350 °C and kept for 120 min under an Ar flow, and NaH_2_PO_2_ was put in the Ar inlet. CoP/MXene, Cu_0.05_Co_0.95_P/MXene, and Cu_0.15_Co_0.85_P/MXene catalyst were obtained using the same method.

### Synthesis of S/Cu*_x_* Co_1−_ *_x_* P/MXene

The S/Cu*_x_* Co_1−_ *_x_* P/MXene composites were fabricated by mixing Cu*_x_* Co_1−_ *_x_* P/MXene and sulfur (the mass ratio was 2:8), and then heating at 155 °C for 10 h in a sealed container filled with argon gas. The sulfur content in S/Cu*_x_* Co_1−_ *_x_* P/MXene was determined by TGA, which was 78.6 wt% (Figure S19, Supporting Information). For comparison, S/MXene composites were also prepared by using the same method.

### Synthesis of Dense S/Cu*_x_* Co_1−_ *_x_* P/MXene Monolith

The dense S/Cu_0.1_Co_0.9_P/MXene monolith was synthesized by forming and drying the S/Cu_0.1_Co_0.9_P/MXene hydrogel via using a small amount of GO (≈15 wt%) as a “self‐assembly agent.” First, 100 mg S/Cu*_x_* Co_1−_ *_x_* P/MXene composites were dispersed into ethanol by ultrasound. After 2 h, 15 mL of GO suspension (GO was ≈17.6 mg) was added into the above solution and stirred thoroughly for at least 30 min. Then, the mixed solution was transferred into a Para polyphenyl (PPL) autoclave, and was heated to 180 °C for 20 h to obtain an S/Cu_0.1_Co_0.9_P/MXene hydrogel. Finally, the as‐prepared S/Cu*_x_* Co_1−_ *_x_* P/MXene hydrogel was dried at 40 °C to form a dense S/Cu_0.1_Co_0.9_P/MXene monolith.

### Statistical Analysis

The charge/discharge performance and cycle stability of the cells were tested on LAND CT3001A battery test systems (Wuhan, China). The cycling stability data were obtained from a single cell in the parallel experiments. CV and EIS measurements (Amplitude = 5 mV; Frequency = 0.01 Hz–100 kHz) were conducted on electrochemical workstation (Eco Chemie Autolab B.V., Metrohm, Switzerland). The above data were obtained by direct testing. The calculation method of the obtained data is detailed in Supporting Information, and the processing softwares were used by the commercial Origin and PowerPoint.

## Conflict of Interest

The authors declare no conflict of interest.

## Supporting information

Supporting Information

## Data Availability

Research data are not shared.
