# Supplementary material for: Unraveling the Atomic‐Level Manipulation Mechanism of Li2S Redox Kinetics via Electron‐Donor Doping for Designing High‐Volumetric‐Energy‐Density, Lean‐Electrolyte Lithium–Sulfur Batteries
Source: Adv Sci (Weinh). 2022 Oct 6;9(33):2204192. doi: 10.1002/advs.202204192 (PMC9685476; doi:10.1002/advs.202204192)
Supplement: Supplementary file 1 — Supporting Information [file ADVS-9-2204192-s001.pdf]

Supporting Information

Unraveling atomic-level manipulation mechanism of Li_2S redox kinetics *via* electron-donor doping for designing high-volumetric-energy-density, lean-electrolyte Li-S batteries

*Jiongwei Shan[1], Wei Wang[1], Bing Zhang[1], Xinying Wang, Weiliang Zhou, Liguo Yue, and Yunyong Li**

J. Shan, W. Wang, B. Zhang, X. Wang, W. Zhou, L. Yue, Prof. Y. Li

School of Materials and Energy, Guangdong University of Technology, No. 100 Waihuan Xi Road, Guangzhou Higher Education Mega Center, Guangzhou 510006, China

*E-mail: yyli@gdut.edu.cn (Y.Y. Li). Tel: (+8620)-39322570, Fax: (+8620)-39322570.

[1]These authors contributed equally to this work.

Section S1. Electrode fabrication and measurements

S1.1 Electrode fabrication

S1.1.1 For the fabrication of S/Cu_xCo_{1-x}P/MXene composite cathode. S/Cu_xCo_{1-x}P/MXene, acetylene black, and polyvinylidene fluoride (PVDF) (the mass ratio is 7.5: 1.5: 1) were mixed and ground, and then added to N-methyl-2-pyrrolidone (NMP) to form a homogeneous slurry, and then, it was coated on an Al foil. For comparison, the S/MXene electrode was also prepared by the identical route. The sulfur mass loading of the cathode was ~1.3 mg cm^{-2}.

S1.1.2 For the fabrication of dense S/Cu_xCo_{1-x}P/MXene monolith cathode. The dense S/Cu_xCo_{1-x}P/MXene monolith cathodes were prepared by mixed dense S/Cu_xCo_{1-x}P/MXene monolith and PVDF with a mass ratio of 9:1 in NMP solvent (**here, no using conductive additive**), and then they were respectively covered on Al foil or carbon-coated Al foil (thick dense sulfur cathodes). The dense S/Cu_xCo_{1-x}P/MXene monolith cathodes with the sulfur loading of 1.3, 5.1, and 9.1 mg cm^{-2} were prepared by controlling the coating thickness.

S1.2 Electrochemical measurement.

The S/Cu_xCo_{1-x}P/MXene, S/MXene, and dense S/Cu_xCo_{1-x}P/MXene monolith cathodes, PP separator (Celgard 2400), lithium metal foil (anode) and lithium bis ((trifluoromethyl) sulfonyl) azanide (LiTFSI) (1.0 M LiTFSI in DME: DOL = 1:1 Vol% with 1.0 wt% $LiNO_3$) were packed into the cell case in a glovebox filled with Ar (<0.1 ppm H_2O and O_2). And the E/S ratio is ~15.0 µL mg^{-1} for the routine tests, whereas the E/S ratios of 5.0 or 3.5 µL mg^{-1} was adopted for the lean electrolyte, respectively.

The cycle performance of the cells was tested on battery test system (LANHE CT3001A) at a potential range of 1.7 to 2.8 V, Cyclic voltammetry (CV) curves and EIS spectra were obtained on electrochemical workstation (Metrohm Autolab) at the scan rates of 0.1 to 10 mV s^{-1}, and with the frequency range of 0.01 Hz to 100 kHz, respectively.

S1.3. Characterization of polysulfides/Li_2S redox kinetics.

S1.3.1 Preparation of lithium polysulfides electrolyte (Li_2S_6 and Li_2S_8 solutions). Li_2S_6 and Li_2S_8 electrolyte/solution can be synthesized by reacting Li_2S and S in electrolyte (1.0 M LiTFSI in DME: DOL = 1:1 Vol%) under continuous stirring for 10 h at 60~80 °C, and the

molar ratios of Li_2S and S were 1:5 (for Li_2S_6) and 1:7 (for Li_2S_8), respectively. After Li_2S and S were completely dissolved, and it was kept in a glove box for later use.

S1.3.2 Static adsorption test for polysulfides. The same amount of $Cu_xCo_{1-x}P$/MXene and MXene (20 mg) were immersed in the as-prepared Li_2S_6 solution (3 mL, from ***S1.3***). The optical image showed that the color faded speed of the electrolyte solution reflects the adsorption capacity of $Cu_xCo_{1-x}P$/MXene and MXene. The Li_2S_6 solutions after fully adsorbed were analyzed by UV-vis absorption spectra, and the intensity of signal peaks showed the content of Li_2S_6 in the solution. Simultaneously, the sediment after Li_2S_6 adsorption ($Cu_xCo_{1-x}P$/MXene- Li_2S_6) were carried out XPS characterization.

S1.3.3 Li_2S nucleation and dissolution measurements. For the Li_2S nucleation measurements, the cells were assembled by using fresh $Cu_xCo_{1-x}P$/MXene electrode as the cathode, Li foil as the anode, and Li_2S_8 solution (1.0 M lithium LiTFSI/0.1 M $LiNO_3$), respectively. First, the cells were galvanostatically discharged at 0.112 mA until the potential was less than 2.11 V. Then, they were discharged potentiostatically at 2.11 V until the current was in a steady state to ensure that Li_2S_8 was sufficiently converted to Li_2S (20000~40000 s). For the Li_2S dissolution measurements, the newly assembled cells were discharged galvanostatically to 1.7 V at a low current of 0.01 mA to ensure that polysulfides were fully converted to Li_2S, they were charged potentiostatically at 2.35 V until the current was in a steady state again (lower than 10^{-5} A). The nucleation and dissolution capacity of Li_2S can be approximated by the integral area of the curves.

S1.3.4 Symmetric cells for liquid-liquid reaction kinetics. The symmetric cells were assembled using $Cu_xCo_{1-x}P$/MXene electrode as both the cathode and anode, and Li_2S_6 electrolyte (1.0 M lithium LiTFSI/0.1 M $LiNO_3$) was used as the electrolyte. The CV curves of the symmetric cells was tested on Metrohm Autolab at different scan rates of 1-10 mV s^{-1} (potential range of -1.0~1.0 V). And the activity of liquid-liquid conversion can be delivered from the CV curves obtained.

Section S2. Materials Characterization Method and DFT Simulation.

S2.1. Characterization Method.

In the work, the compositions of the obtained products were characterized on powder X-ray diffraction (XRD) (Rigaku Dmaxrc diffractometer with Cu Kα radiation) operated at 40 kV voltage and 40 mA current. The microstructures and elemental mapping distribution of the samples were characterized by SU 8010 field emission scanning electron microscopy (SEM) and transmission electron microscopy (TEM, FEI, Talos F200S) under an accelerating voltage of 200 kV. The X-ray photoelectron spectra (XPS) were measured on an ESCALAB 250 spectrometer to analyse the surface chemical property. The thermogravimetric analysis (TGA, NETZSCHSTA 409 PC) was used to calculate the sulfur content of $S/Cu_xCo_{1-x}P$/MXene in N_2 with a heating rate of 10 ^oC min^{-1} from 30 to 700 ^oC. The pore size distribution and specific surface area of samples were conducted *via* Brunauer-Emmett-Teller (BET, ASAP 2460). Various host materials and sulfur hybrid materials were outgassed at 120 and 80 ^oC in N_2 for 10 h before the test, respectively. UV-vis spectra were collected by ultraviolet-visible-near infrared spectrophotometer (UV-3600 plus).

S2.2. Density Functional Theory (DFT) Calculation Method.

First-principle DFT calculations are performed by Vienna Ab initio Simulation Package(VASP)[S1] with the projector augmented wave (PAW) method[S2]. The exchange-functional is treated using the generalized gradient approximation (GGA) of Perdew-Burke-Ernzerhof (PBE) functional[S3]. The energy cutoff for the plane wave basis expansion was set to 450 eV and the force on each atom less than 0.03 eV/Å was set for convergence criterion of geometry relaxation. 15 Å vacuum was added along the z direction in order to avoid the interaction between periodic structures. The Brillouin zone integration was sampled by single Γ point. The self-consistent calculations apply a convergence energy threshold of 10^{-4} eV. The DFT-D3 method was employed to consider the van der Waals interaction[S4]. Transition state searching were calculated using the climbing-image nudged

clastic band (CI-NEB) method[S5].

The adsorption energy of Li_2S_x was calculated according to

$E_{ads} = E_{total} - E_{sub} - E_{Li2Sx}$

Where E_{total} is the total energy of the Li_2S_x adsorbed systems, E_{sub} and E_{Li2Sx} are the energies of the substrate and the isolated Li_2S_x molecule, respectively.

Section S3. Calculation methods and details for diffusion coefficient, activation energy, electrical conductivity and apparent density of dense sulfur monolith, and compacted density and volumetric capacity of dense sulfur cathode.

S3.1. Diffusion coefficient calculation

The diffusion coefficient of Li ions can be calculated according to the *Randles-Sevick* equation on the basis of the above CV results

$$I_p = 2.69 \times 10^5 n^{1.5} A\, D_{Li+}^{0.5} C_{Li+} v^{0.5}$$

where I_p is the peak current and n indicates the number of transferred electrons in the reaction (n = 2 for LSBs), A represents the area of the electrode (1.13 cm^2 here), C_{Li+} means the concentration of the lithium ions in the electrolyte, v is the scanning rate ($V\ s^{-1}$).

S3.2. Calculation of Activation Energy

Activation energy (E_a) can be fitted to the Arrhenius equation

$$j \propto A \times e^{-Ea/RT},$$

where E_a is the activation energy, R is the gas constant (8.314 J $(mol\ K)^{-1}$), A is a pre-exponential factor, and T is temperature.

S3.3. Calculation Methods for apparent density of dense S/Cu$_{0.1}$Co$_{0.9}$P/MXene monolith and compacted density of dense sulfur cathode.

For the convenience of calculation, the diameter (d) and height (h) of cylinders after shrinkage were ~0.35 cm and ~0.6 cm, respectively. The mass (m) of the dense $S/Cu_{0.1}Co_{0.9}P$/MXene monolith is 112.5 mg. For comparison, the diameter (~1.2 cm) and height (2.05 cm) of hydrogel before shrinkage were also estimated. Therefore, the apparent density (ρ_a) of the dense $S/Cu_{0.1}Co_{0.9}P$/MXene monolith can be calculated according to the following equation:

$$V = \pi \times (\frac{d}{2})^2 \times h$$

$$\rho_a = \frac{m}{V} = \frac{112.5\ mg}{\pi \times 0.175^2 \times 0.6\ cm^3} \approx 1.95\ g\ cm^{-3}$$

In addition, it is calculated that the volume of hydrogel before and after shrinkage is 2.32 and 0.0577 cm^3, respectively, and the dense monolith after air drying was shrank to 1/40 of parent hydrogel in volume. The weight of the dense $S/Cu_{0.1}Co_{0.9}P$/MXene cathode is ~9.65 mg, and its thickness is 44.2 μm measured by SEM image (see **Figure S25**). Therefore, the compacted density is calculated, which is 1.93 g cm^3 (here, the electrode area is ~1.13 cm^{-2}).

S3.4. Calculation of the electrical conductivity for dense sulfur monolith

The electrical conductivity of $S/Cu_{0.1}Co_{0.9}P$/MXene monolith can be calculated according to the following equation:

$$\sigma = \frac{1}{\rho}$$

$$\rho = R \times \frac{S}{L}$$

$$\sigma = \frac{L}{RS} = \frac{0.6\ cm}{2.2\Omega \times\ (\pi \times 0.175^2\ )\ cm^2} = 283\ S\ m^{-1}$$

where σ, ρ, L, R, and S represent the electrical conductivity, resistivity, length of the monolith, resistance, and cross-sectional area, respectively.

S3.5. Calculation of volumetric capacity.

The volumetric capacity (C_v) of the dense $S/Cu_{0.1}Co_{0.9}P$/MXene monolith can be calculated according to the following equation:

$$C_g = C_{gs} \times 59.76\%$$

$$\rho_{(cathode)} = m_{cathode}/V_{cathode}$$

$$C_v = C_g \times \rho_{(S)}$$

C_g, C_{gs}, $\rho_{(cathode)}$, $m_{cathode}$, and $V_{cathode}$ represent the gravimetric capacity (based on the whole sulfur cathode), gravimetric capacity (based on the sulfur), compacted density of the cathode, mass of the cathode (~ 9.65 mg), and volume of the cathode (~0.005 cm^3), respectively. The gravimetric capacity of the dense $S/Cu_{0.1}Co_{0.9}P$/MXene monolith cathode with the sulfur

loading of 5.1 mg cm^{-2} or 1.3 mg cm^{-2} is 1110 or 1443 mAh g^{-1}, respectively, and the sulfur content in the whole cathode is 59.76 wt% (the sulfur content in S/$Cu_{0.1}Co_{0.9}P$/MXene monolith is 66.4 wt%, and the binder is 10 wt%, no using conductive additive). Therefore, the calculated C_v of the dense S/$Cu_{0.1}Co_{0.9}P$/MXene monolith cathode are 1280 Ah L^{-1} and 1664 Ah L^{-1} based on the total volume of the whole sulfur cathode, respectively (here $\rho_{(cathode)}$ = 1.93 g cm^3).

Additional Results:

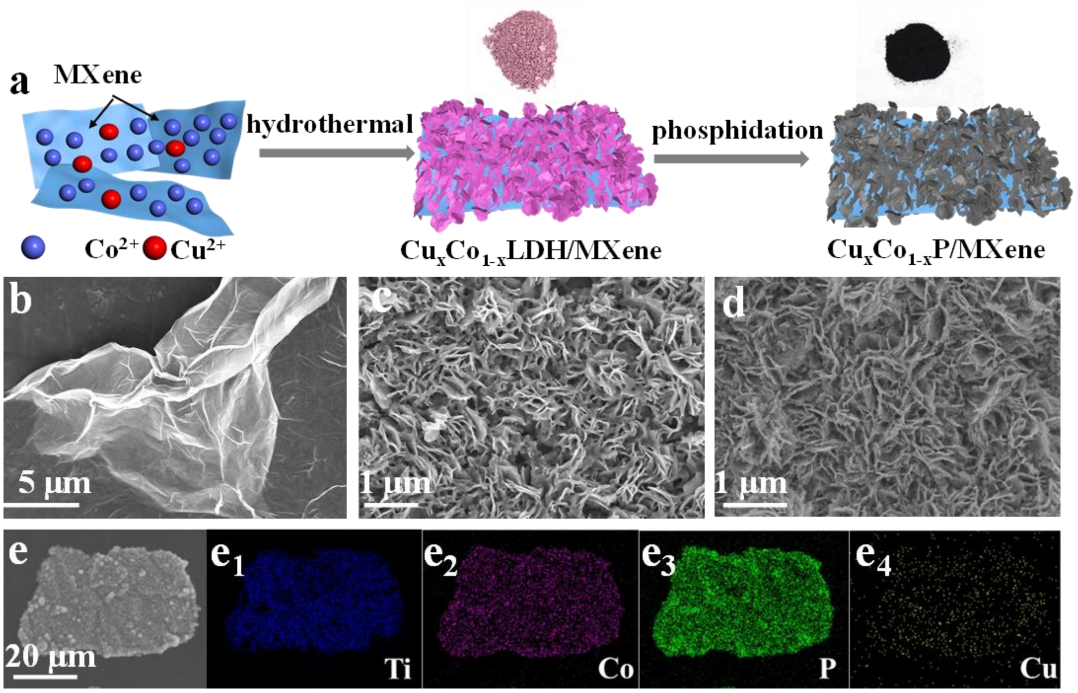

Figure S1. a) Schematic illustration for the synthesis of $Cu_xCo_{1-x}P$/MXene. SEM images of b) MXene, c) Cu_xCo_{1-x}LDH/MXene, and d-e) $Cu_{0.1}Co_{0.9}P$/MXene and corresponding EDX elemental mapping showing the homogeneous distribution of Ti, Co, P, and Cu elements.

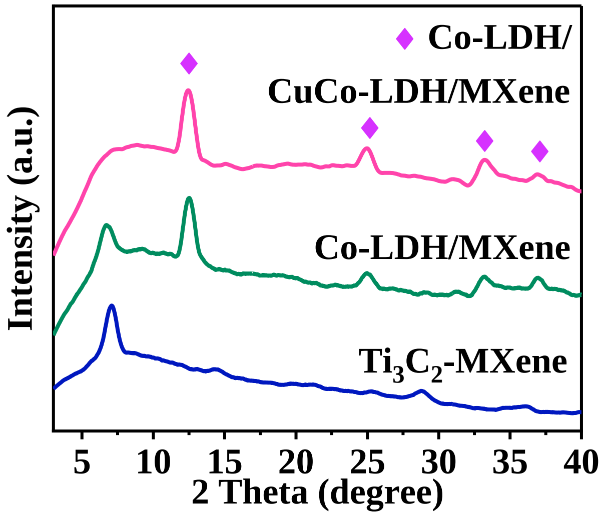

Figure S2. XRD patterns of Ti_3C_2-MXene, Co-LDH/MXene, and CuCo-LDH/MXene.

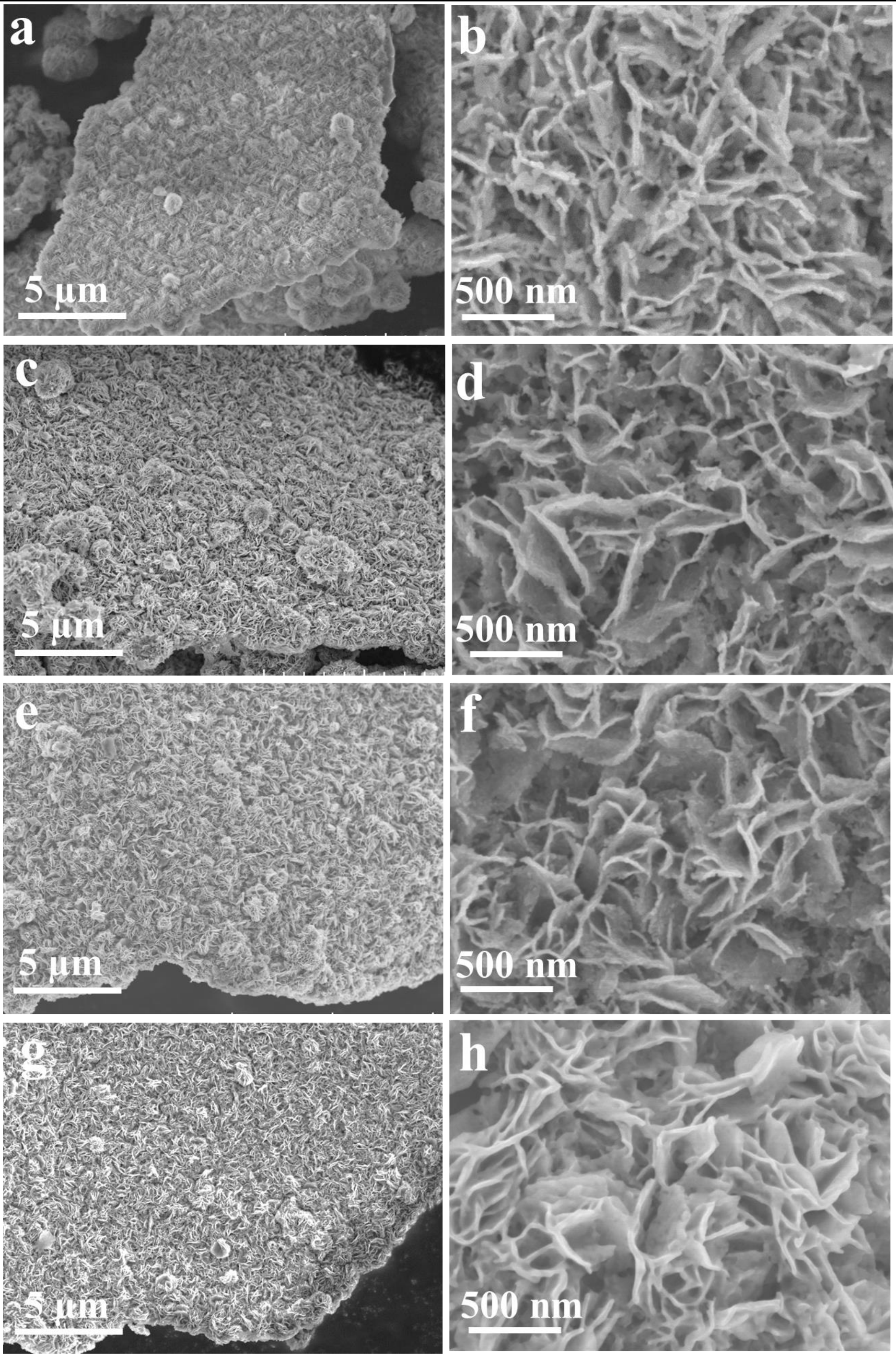

Figure S3. SEM images of a, b) CoP/MXene, c, d) $Cu_{0.05}Co_{0.95}P$ P/MXene, e, f) $Cu_{0.1}Co_{0.9}P$ /MXene, and g, h) $Cu_{0.15}Co_{0.85}P$/MXene.

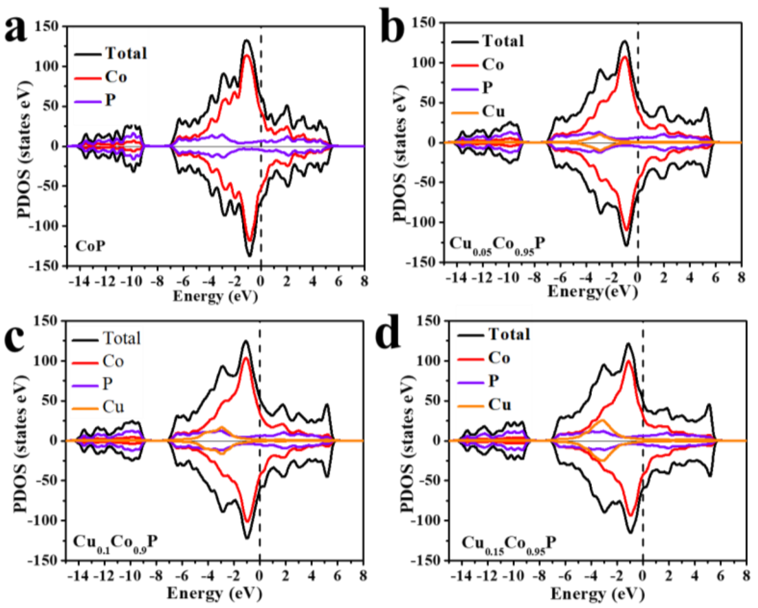

Figure S4. The density of states of a) CoP, b) $Cu_{0.05}Co_{0.95}P$, c) $Cu_{0.1}Co_{0.9}P$ and d) $Cu_{0.15}Co_{0.85}P$. The number of electronic states at Fermi level indicates the metal property.

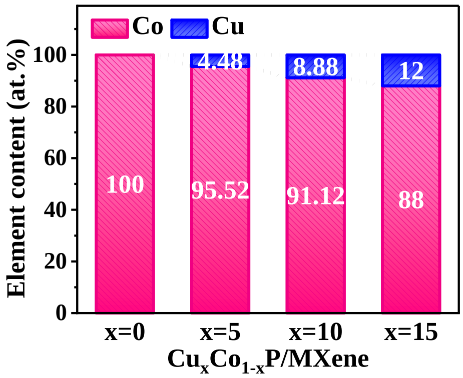

Figure S5. Elemental content analysis using ICP-OES for $Cu_xCo_{1-x}P$/MXene.

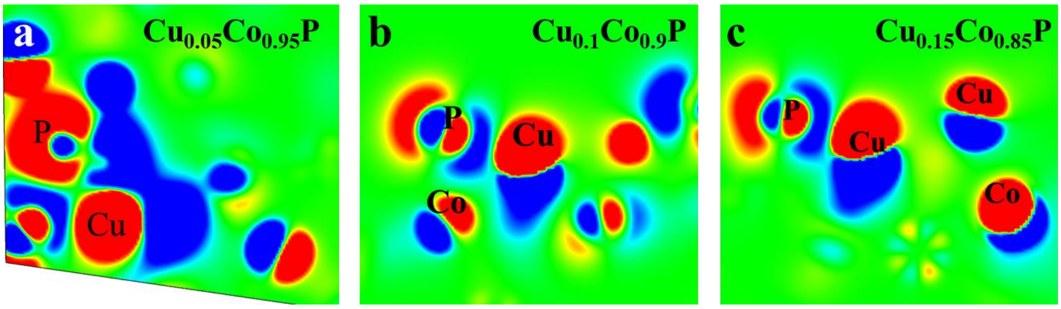

Figure S6. The difference in charge density of a) $Cu_{0.05}Co_{0.95}P$, b) $Cu_{0.1}Co_{0.9}P$, and c) $Cu_{0.15}Co_{0.85}P$ (isosurface level is set to 0.004 e Å^{-3}) (red and blue represent the electron

accumulate and depletion regions, respectively).

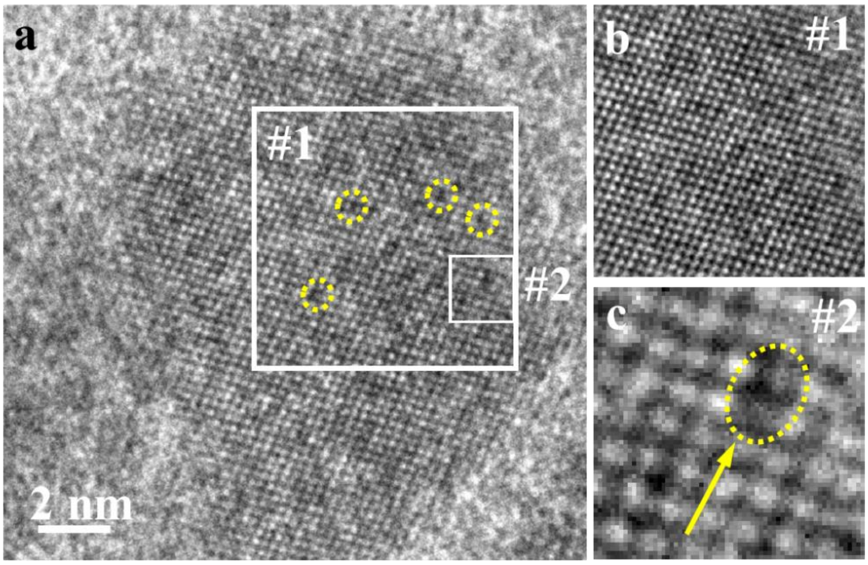

Figure S7. HR-TEM images of $Cu_{0.1}Co_{0.9}P$/MXene.

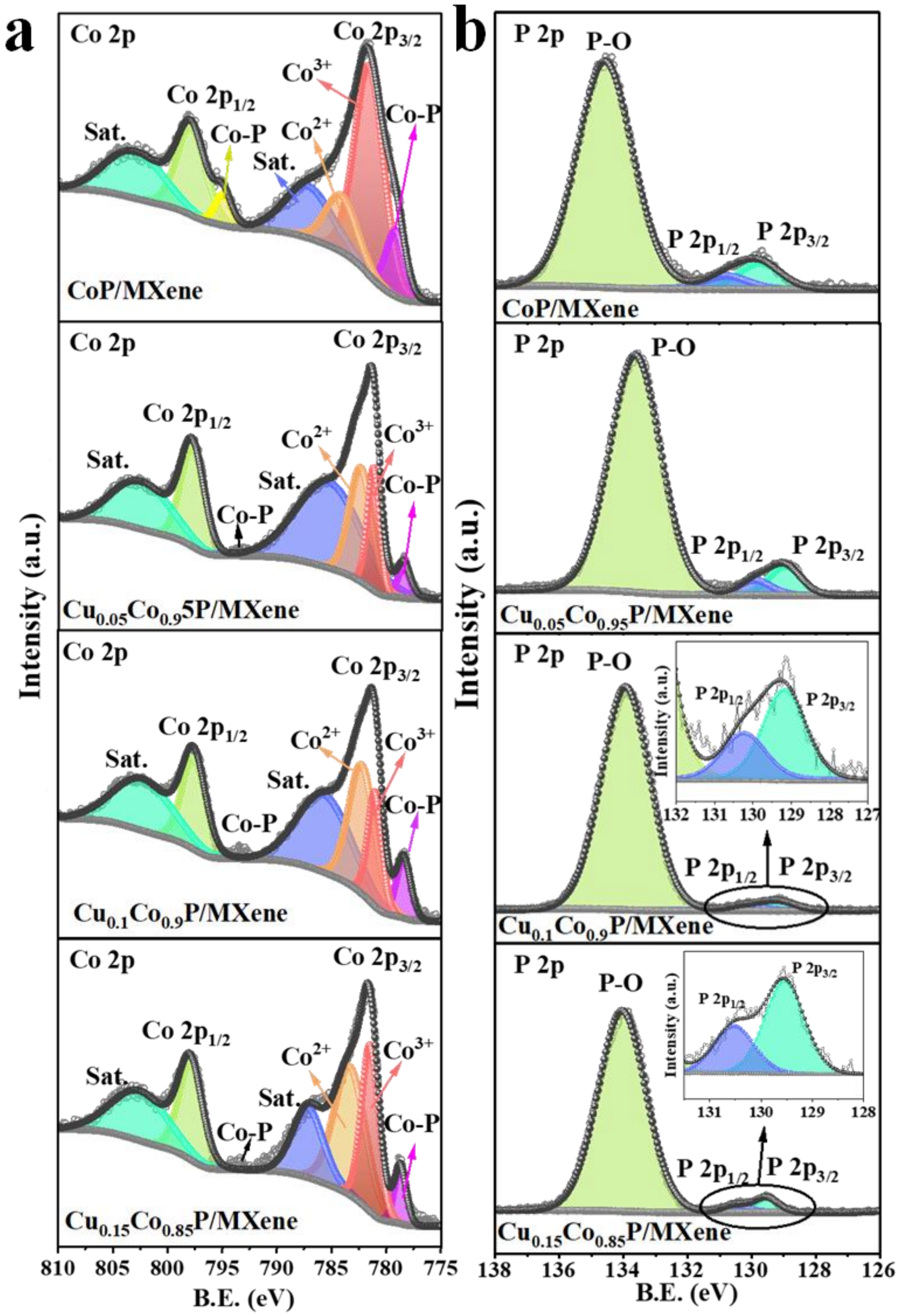

Figure S8. High-resolution XPS spectra of a) Co 2p and b) P 2p for $Cu_xCo_{1-x}P$/MXene.

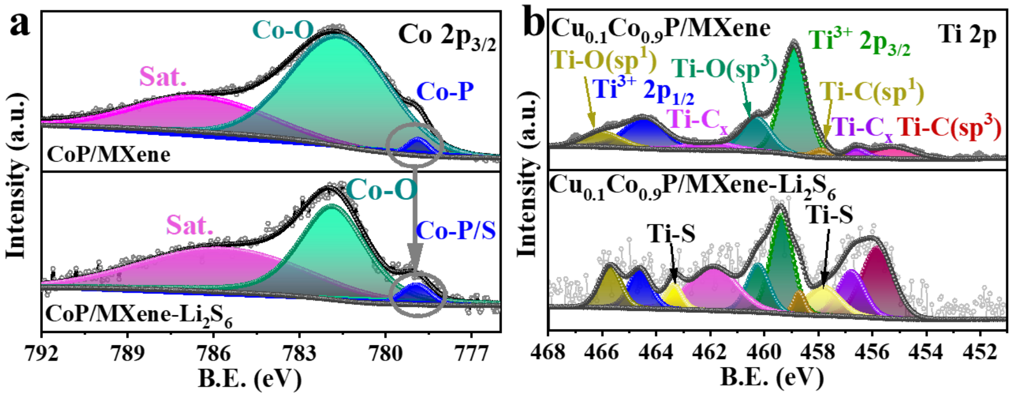

Figure S9. **High-resolution XPS spectra.** a) Co $2p_{3/2}$ spectra of CoP/MXene and CoP/MXene-Li_2S_6. b) Ti 2p spectra of $Cu_{0.1}Co_{0.9}P$/MXene and $Cu_{0.1}Co_{0.9}P$/MXene-Li_2S_6.

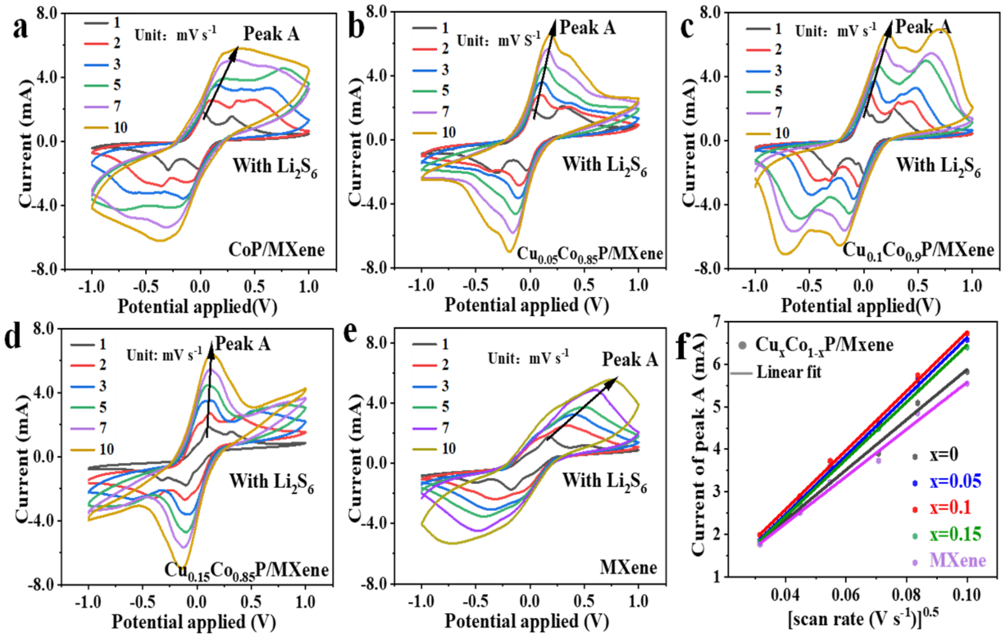

Figure S10. CV curves of a) CoP/MXene, b) $Cu_{0.05}Co_{0.95}P$/MXene, c) $Cu_{0.1}Co_{0.9}P$/MXene, d) $Cu_{0.15}Co_{0.85}P$/MXene, and e) MXene in the non-faradic current range under different scan rates (10-100 mV s^{-1}). f) Relation of peak currents with different scan rates (1, 2, 3, 5, 7, and 10 mV s^{-1}).

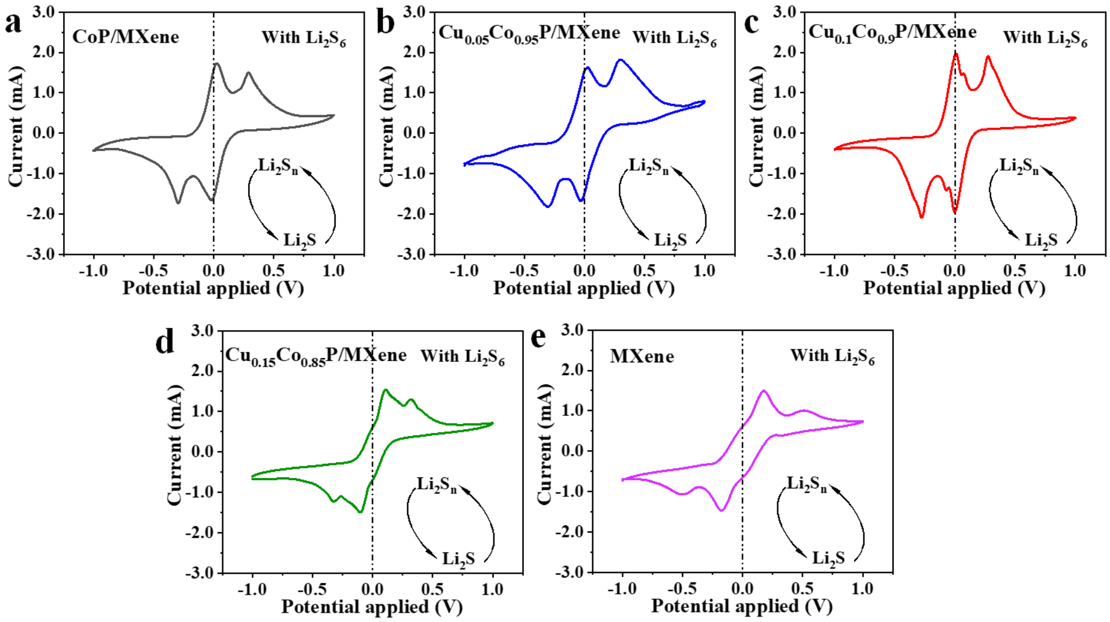

Figure S11. CV curves of a) CoP/MXene, b) $Cu_{0.05}Co_{0.95}$P/MXene, c) $Cu_{0.1}Co_{0.9}$P/MXene, d) $Cu_{0.15}Co_{0.85}$P/MXene, and e) MXene at scan rates of 1mV s^{-1}.

Nucleation of Li_2S.

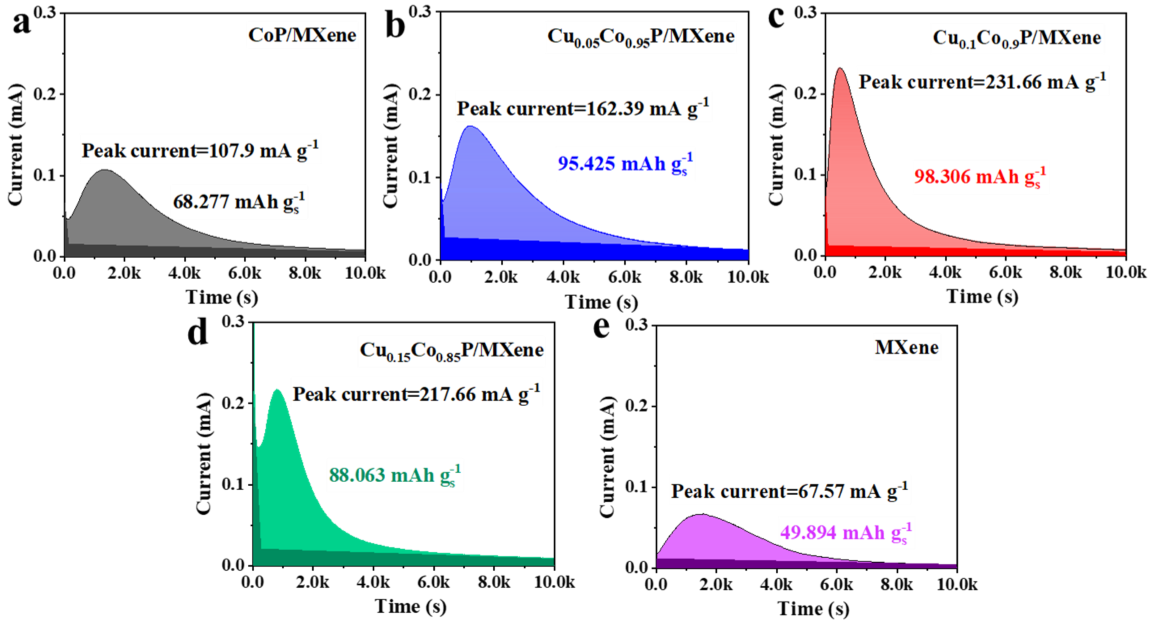

Figure S12. Potentiostatic discharge profiles of a) CoP/MXene, b) $Cu_{0.05}Co_{0.95}$P/MXene, c) $Cu_{0.1}Co_{0.9}$P/MXene d) $Cu_{0.15}Co_{0.85}$P/MXene, and e) MXene electrodes at 2.11 V, respectively.

Decomposition of Li_2S.

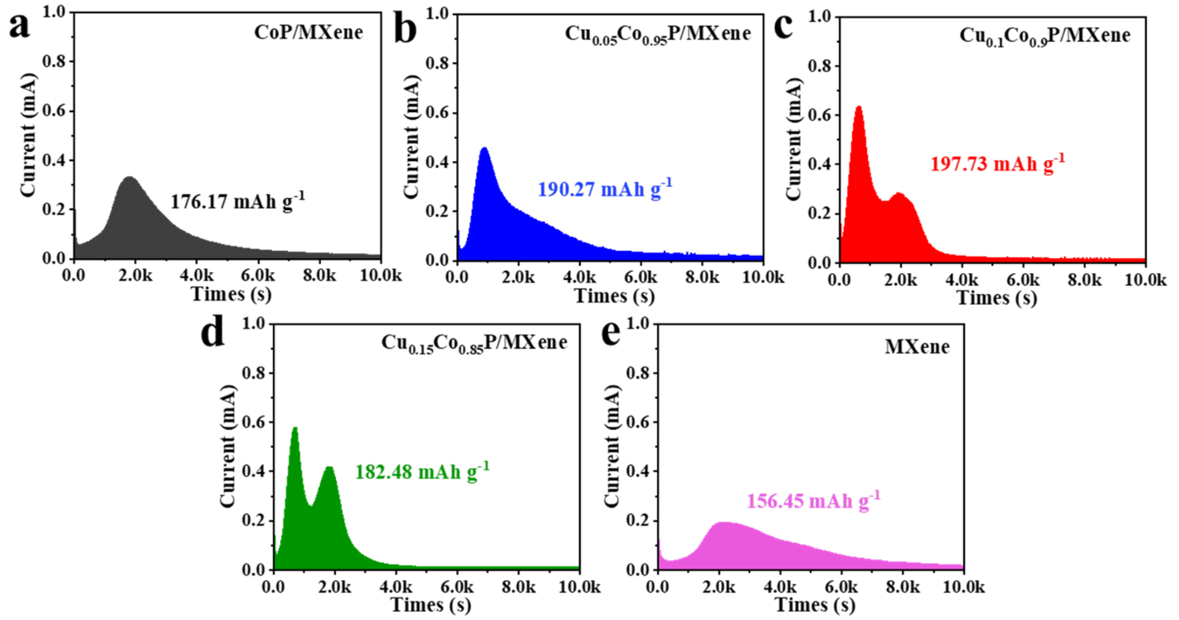

Figure S13. Potentiostatic charge profiles of a) CoP/MXene, b) $Cu_{0.05}Co_{0.95}P$/MXene, c) $Cu_{0.1}Co_{0.9}P$/MXene d) $Cu_{0.15}Co_{0.85}P$/MXene, and e) MXene electrodes at 2.35 V, respectively.

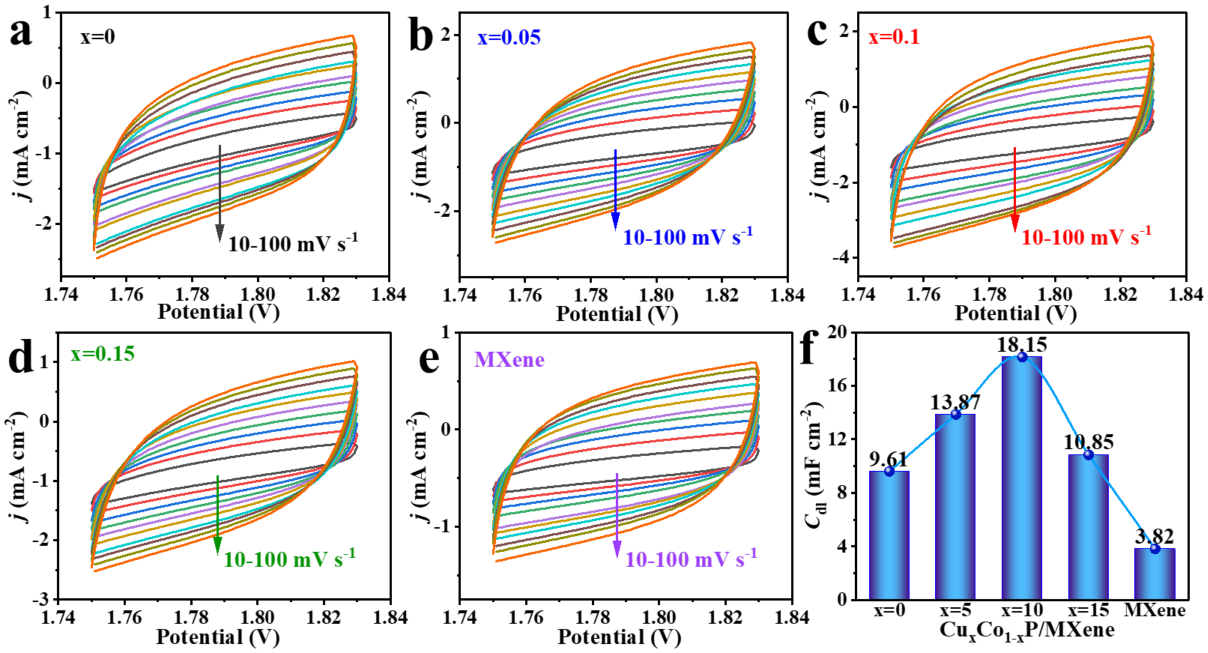

Figure S14. CV curves of a) CoP/MXene, b) $Cu_{0.05}Co_{0.95}P$/MXene, c) $Cu_{0.1}Co_{0.9}P$/MXene d) $Cu_{0.15}Co_{0.85}P$/MXene and e) MXene in the non-faradic current range at various scan rates (10-100 mV s^{-1}). f) The double layer capacitance of $Cu_xCo_{1-x}P$/MXene.

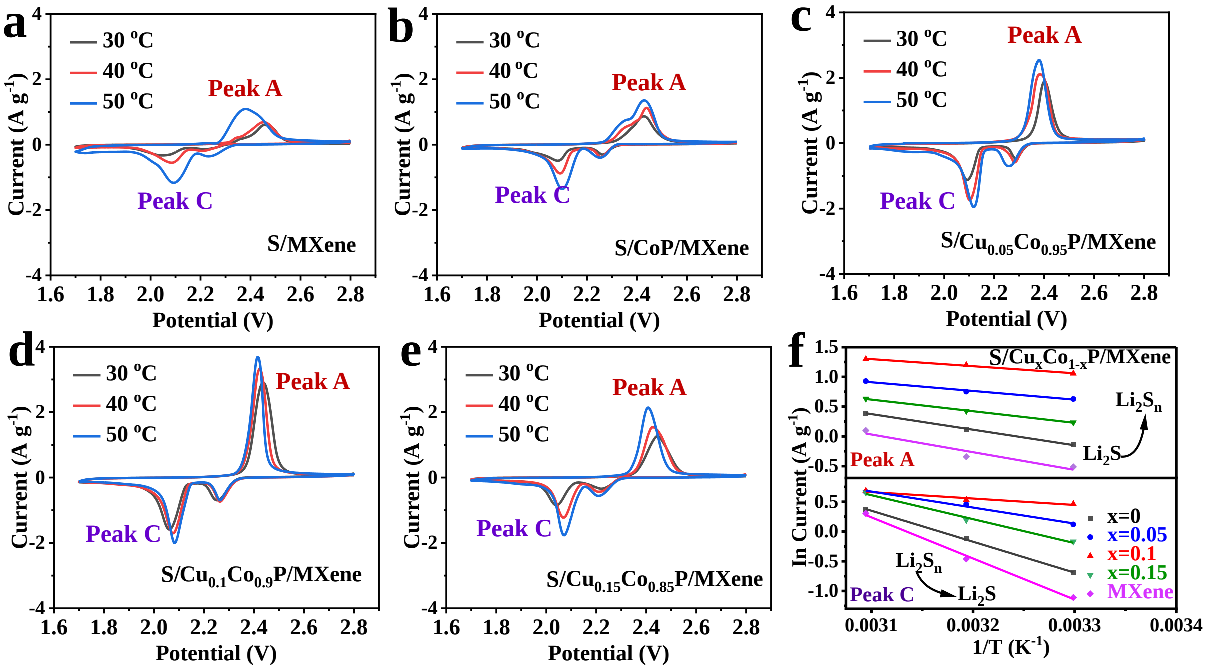

Figure S15. The CV curves at different temperatures of 30, 40, and 50 °C for the cells using a) S/MXene, b) S/CoP/MXene, c) $S/Cu_{0.05}Co_{0.95}P/MXene$, d) $S/Cu_{0.1}Co_{0.9}P/MXene$, and e) $S/Cu_{0.15}Co_{0.85}P/MXene$ as cathodes and Li metal as the anode. f) Relation of Li_2S and Li_2S_n interconversion rates with respect to temperatures in Li-S cells with $S/Cu_xCo_{1-x}P/MXene$ and S/MXene.

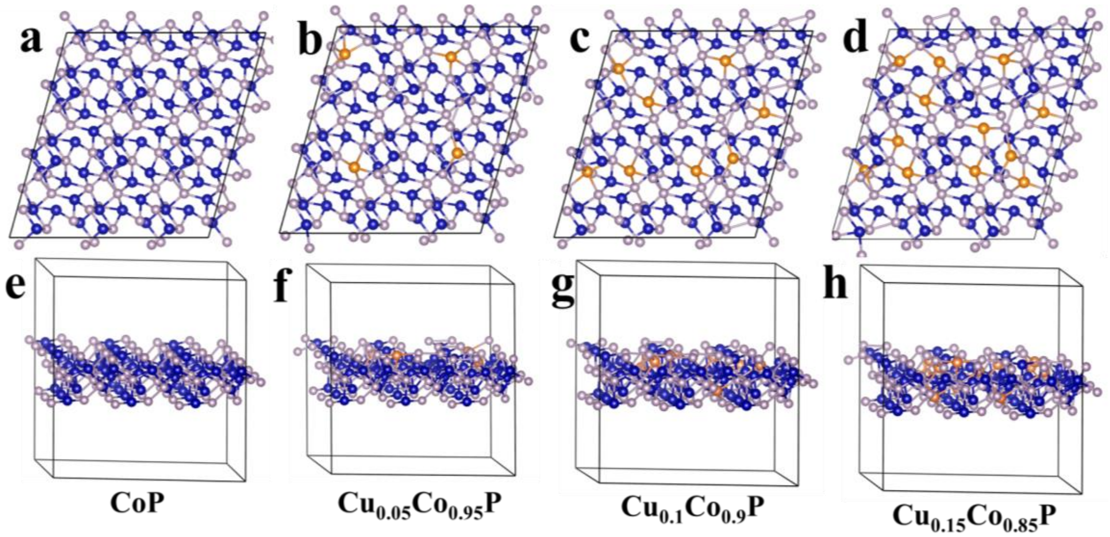

Figure S16. a-d) Top views and e-h) side views of the theoretical calculation models for $Cu_xCo_{1-x}P$ (X= 0, 0.05, 0.1, and 0.15).

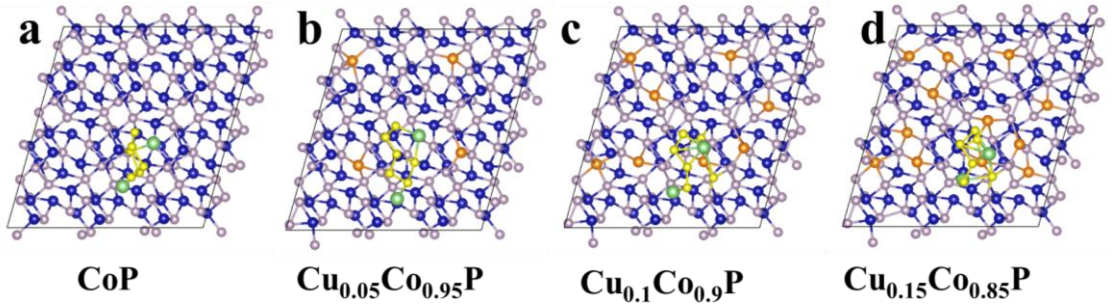

Figure S17. Top views of the Li_2S_6 adsorbed on the surface of a) CoP, b) $Cu_{0.05}Co_{0.95}P$, c) $Cu_{0.1}Co_{0.9}P$, and d) $Cu_{0.15}Co_{0.85}P$.

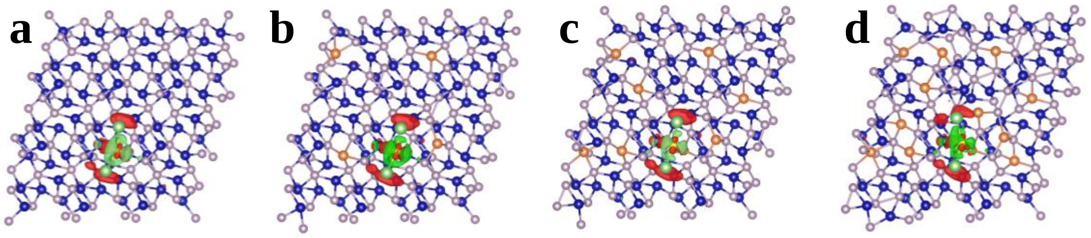

Figure S18. Top views of the difference in charge density of a) CoP-Li_2S, b) $Cu_{0.05}Co_{0.95}P$-Li_2S, c) $Cu_{0.1}Co_{0.9}P$-Li_2S, and d) $Cu_{0.15}Co_{0.85}P$-Li_2S.

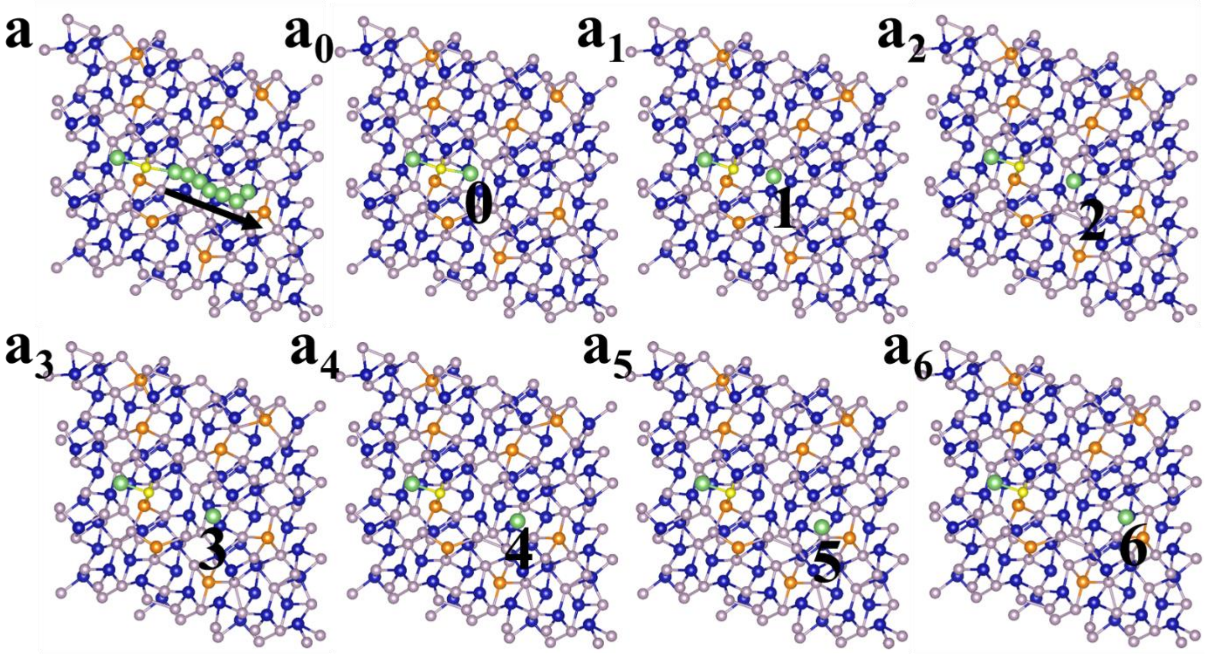

Figure S19. The diffusion path of Li_2S on the surface of $Cu_{0.1}Co_{0.9}P$. a) Total location moving. a_0-a_6) Initial state to final state.

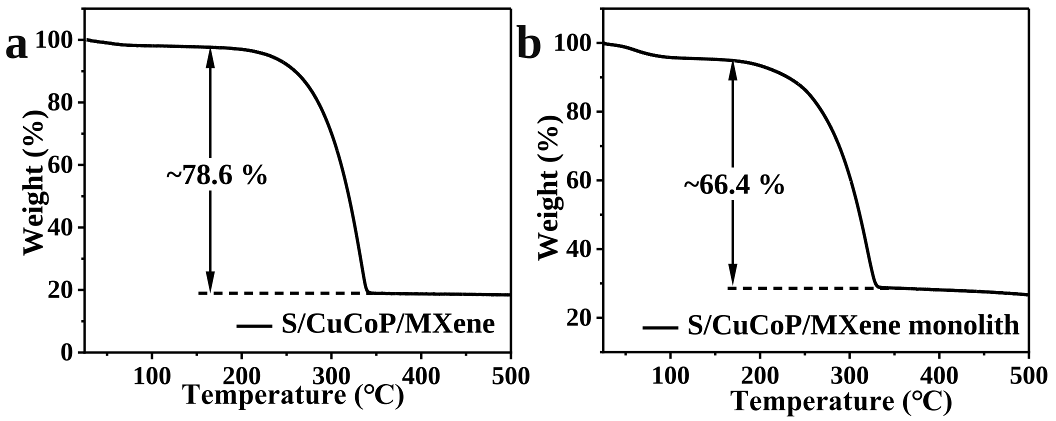

Figure S20. Thermogravimetric analysis (TGA) curves of a) S/Cu_xCo_{1-x}P/MXene and b) dense S/$Cu_{0.1}Co_{0.9}$P/MXene monolith in N_2.

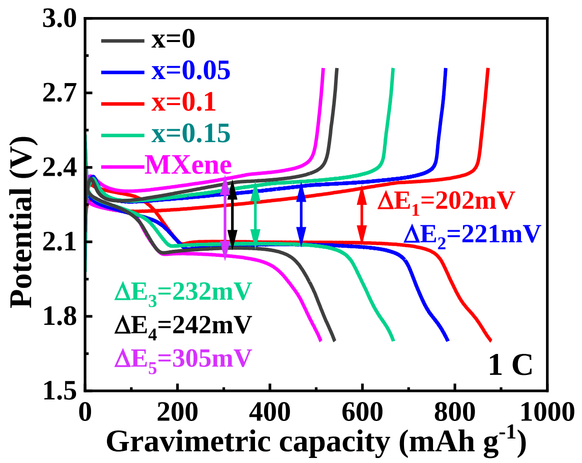

Figure S21. Galvanostatic charge/discharge profiles of Cu_xCo_{1-x}P/MXene at 1 C.

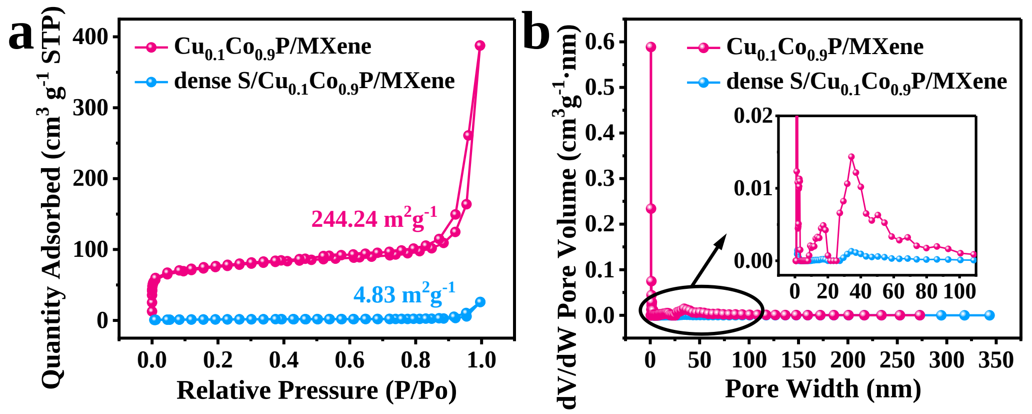

Figure S22 a) N_2 adsorption/desorption isotherms and b) pore size distributions of $Cu_{0.1}Co_{0.9}P$/MXene composite and dense S/$Cu_{0.1}Co_{0.9}P$/MXene monolith.

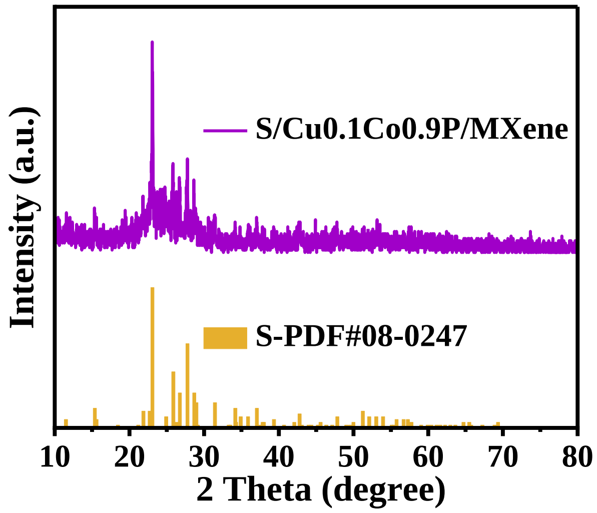

Figure S23. XRD pattern of dense S/$Cu_{0.1}Co_{0.9}P$/MXene monolith.

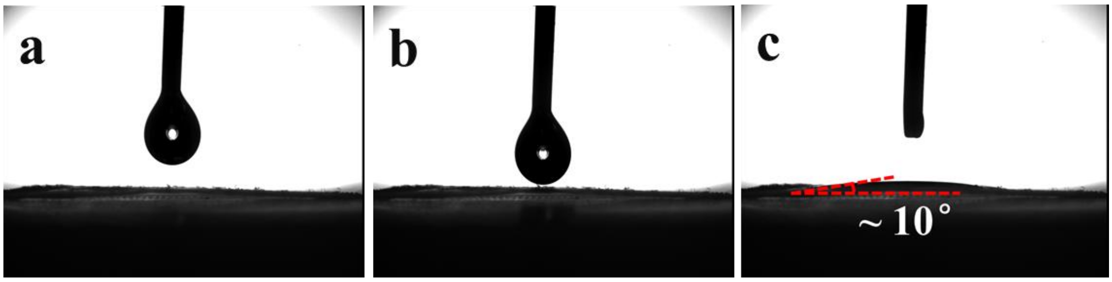

Figure S24 Optical pictures of the interface contact between the electrolyte and dense S/$Cu_{0.1}Co_{0.9}P$/MXene monolith particles coated on the surface of Al foil (containing 10 wt% binder).

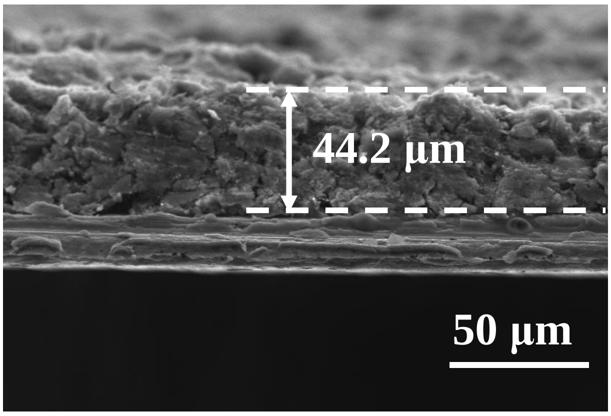

Figure S25. SEM image of a thick and dense S/$Cu_{0.1}Co_{0.9}P$/MXene monolith cathode with the sulfur loading of 5.1 mg cm^{-2}.

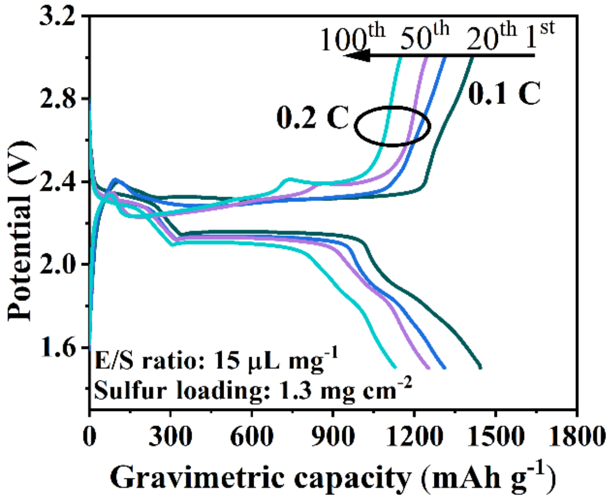

Figure S26. Galvanostatic charge/discharge curves of dense S/$Cu_{0.1}Co_{0.9}P$/MXene monolith cathode with 1.3 mg cm^{-2} sulfur loading in a routine electrolyte.

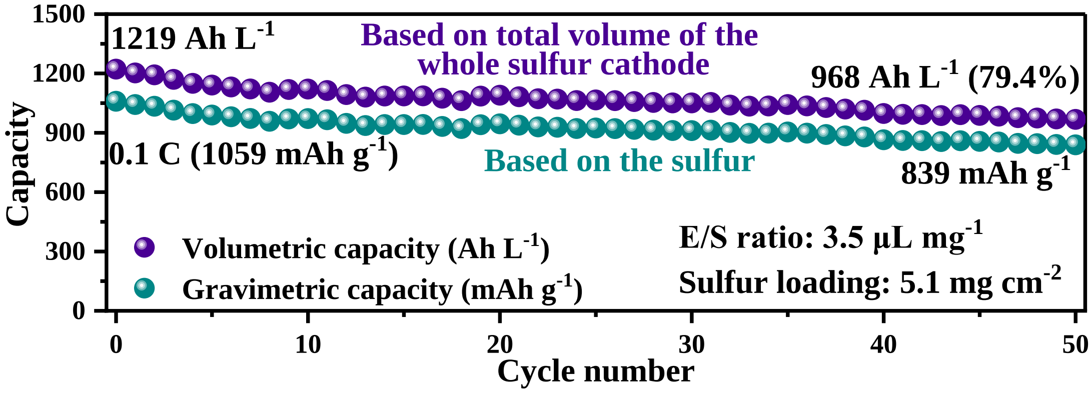

Figure S27. Gravimetric and volumetric capacities of a thick dense S/$Cu_{0.1}Co_{0.9}P$/MXene monolith cathode under a low E/S ratio of 3.5 μL mg^{-1} at 0.1 C.

Table S1. The binding energy of Co^{2+} and $Co^{3+}$and the area ratios of Co^{2+} in Co 2p XPS spectra.

$Cu_xCo_{1-x}P$/MXene	Co^{3+} $2p_{3/2}$ (eV)	Co^{2+} $2p_{3/2}$ (eV)	Content of Co^{2+} (%)
x = 0	781.58	784.17	17.16
x = 0.05	781.07	782.28	26.94
x = 0.1	781.2	782.39	36.01
x = 0.15	781.49	783.36	44.24

Table S2. Performance comparison of the dense S/$Cu_{0.1}Co_{0.9}$P/MXene cathode with recent reported advanced high-density cathodes.

Cathode materials	Material density (g cm^{-3})	Initial gravimetric capacity (mAh g^{-1})	Volumetric capacity (Ah L^{-1})*	Max areal capacity (mAh cm^{-2})	E/S Ratio (μL mg^{-1})	Refs.
**S/$Cu_{0.1}Co_{0.9}$P /MXene**	**1.95**	**1110 (0.1 C)**	**1280**	**8.3**	**5.0†**	**This work**
		**1057 (0.1 C)**	**1219**	**5.40**	**3.5†**	
		**1443 (0.1 C)**	**1664**	**6.36**	**15**	
HDGS-S	1.53	750 (0.1 C)	1148	-	20	[S6]
S@HKUST-$_1$/ CNT	1.41	1263 (0.2 C)	1246	14.7	15	[S7]
rGO-VS_2/S	1.84	1289 (0.1 C)	1328	~2.6	10	[S8]
$NiFe_2O_4$/S	1.33	1288 (0.1 C)	1282	-	20	[S9]
VO_2 HSs@S	1.64	930 (0.1 C)	1084	-	32	[S10]
FeS_2/FeS/S	1.4	1045 (0.1 C)	1422	1.2	20	[S11]
G/CNT	1.64	1128 (0.5 C)	1182	-	20	[S12]
TCD-TCS/S	~1.91	1081 (1 C)	1445	13.7	20	[S13]
S/$NiCo_2O_4$	1.66	1500 (0.1 C)	1307	3.4	25	[S14]
		1253 (0.1 C)	1092	1.5	5.0†	
S/CoOOH	1.26	1199 (0.1 C)	1134	4.35	30	[S15]
		~900 (0.1 C)	1057.7	2.46	8.0†	
S/$La_{0.8}Sr_{0.2}MnO_3$	1.69	1191 (0.1 C)	1299	7.3	10	[S16]
		900 (0.1 C)	973	1.3	5.0†	
S/HEO	1.92	1368 (0.1 C)	1464	4.4	20	[S17]
c-(rGOCoS_2)/S	1.75	908 (1 C)	1240	6.67	15	[S18]
FM@G/87S	1.88	1040 (0.5 C)	1360	5.3	15	[S19]
		905 (0.5 C)	1183	4.62	6†	
CoB/NBC-S	1.58	1309 (0.1 C)	1355	7.59	10	[S20]

*The volumetric capacity was calculated based on the total volume of the whole sulfur cathode.

†Blue highlights represent the electrochemical performance in a lean electrolyte.

References

[S1] G. Kresse and J. Furthüller, *Comp. Mater. Sci.* **1996**, 6, 15.

[S2] P. E. Blochl, *Phys. Rev. B* **1994**, 50, 17953.

[S3] J. P. Perdew, J. A. Chevary, S. H. Vosko, K. A. Jackson, M. R. Pederson, D. J. Singh and C. Fiolhais, *Phys. Rev. B* **1992**, 46, 6671.

[S4] S. Grimme, J. Antony, S. Ehrlich and H. Krieg, *J. Chem. Phys.* **2010**, 132, 154104.

[S5] G. Henkelman, B. P. Uberuaga and H. Jónsson, *J.Chem. Phys.* **2000**, 113, 9901.

[S6] C. Zhang, D. H. Liu, W. Lv, D. W. Wang, W. Wei, G. M. Zhou, S. Wang, F. Li, B. H. Li, F. Kang and Q. H. Yang, *Nanoscale* **2015**, 7, 5592.

[S7] Y. Mao, G. Li, Y. Guo, Z. Li, C. Liang, X. Peng and Z. Lin, *Nat. Commun.* **2017**, 8, 14628.

[S8] Z. Cheng, Z. Xiao, H. Pan, S. Wang and R. Wang, *Adv. Energy Mater.* **2018**, 8, 1702337.

[S9]Z. Zhang, D.-H. Wu, Z. Zhou, G.-R. Li, S. Liu and X.-P. Gao, *Sci. China Mater.* **2018**, 62, 74.

[S10] L. Zhou, L. Yao, S. Li, J. Zai, S. Li, Q. He, K. He, X. Li, D. Wang and X. Qian, *J. Mater. Chem. A* **2019**, 7, 3618.

[S11] K. Xi, D. He, C. Harris, Y. Wang, C. Lai, H. Li, P. R. Coxon, S. Ding, C. Wang and R. V. Kumar, *Adv. Sci.* **2019**, 6, 1800815.

[S12] H. Shi, X. Zhao, Z. S. Wu, Y. Dong, P. Lu, J. Chen, W. Ren, H.-M. Cheng and X. Bao, *Nano Energy* **2019**, 60, 743.

[S13] Z. Xiao, Z. Li, P. Li, X. Meng and R. Wang, *ACS Nano* **2019**, 13, 3608.

[S14] Y. T. Liu, D. D. Han, L. Wang, G. R. Li, S. Liu and X. P. Gao, *Adv. Energy Mater.* **2019**, 9, 1803477.

[S15] Z. Y. Wang, L. Wang, S. Liu, G. R. Li and X. P. Gao, *Adv. Funct. Mater.* **2019**, 29, 1901051.

[S16] Y. T. Liu, S. Liu, G. R. Li, T. Y. Yan and X. P. Gao, *Adv. Sci.* **2020**, 7, 1903693.

[S17] L. Y. Tian, Z. Zhang, S. Liu, G. R. Li and X. P. Gao, *Energy Environ. Mater.* **2021**, 5, 645.

[S18] H. Li, X. Wen, F. Shao, C. Zhou, Y. Zhang, N. Hu and H. Wei, *Chem. Eng. J.* **2021**, 412, 1228562.

[S19] Z. B. Cheng, Y. L. Chen, Y. S. Yang, L. J. Zhang, H. Pan, X. Fan, S. C. Xiang and Z. J. Zhang, *Adv. Energy Mater.* **2021**, 11, 2003718.

[S20] Z. Li, P. Li, X. Meng, Z. Lin and R. Wang, *Adv. Mater.* **2021**, 33, 2102338.
